# Between-occupation differences in work-related COVID-19 mitigation strategies over time: Analysis of the Virus Watch Cohort in England and Wales

**DOI:** 10.5271/sjweh.4092

**Published:** 2023-07-01

**Authors:** Sarah Beale, Alexei Yavlinsky, Susan Hoskins, Vincent Nguyen, Thomas Byrne, Wing Lam Erica Fong, Jana Kovar, Martie Van Tongeren, Robert W Aldridge, Andrew Hayward

**Affiliations:** 1 Centre for Public Health Data Science, Institute of Health Informatics, University College London, UK, NW1 2DA.; 2 Institute of Epidemiology and Health Care, University College London, London, UK, WC1E 7HB.; 3 Division of Population Health, Health Services Research & Primary Care, University of Manchester, Manchester, UK, M13 9NT.

**Keywords:** infection control, infection prevention, occupational health, pandemic, SARS-CoV-2

## Abstract

**Objectives:**

COVID-19 mitigations have had a profound impact on workplaces, however, multisectoral comparisons of how work-related mitigations were applied are limited. This study aimed to investigate (i) occupational differences in the usage of key work-related mitigations over time and (ii) workers’ perceptions of these mitigations.

**Methods:**

Employed/self-employed Virus Watch study participants (N=6279) responded to a mitigation-related online survey covering the periods of December 2020–February 2022. Logistic regression was used to investigate occupation- and time-related differences in the usage of work-related mitigation methods. Participants’ perceptions of mitigation methods were investigated descriptively using proportions.

**Results:**

Usage of work-related mitigation methods differed between occupations and over time, likely reflecting variation in job roles, workplace environments, legislation and guidance. Healthcare workers had the highest predicted probabilities for several mitigations, including reporting frequent hand hygiene [predicted probability across all survey periods 0.61 (95% CI 0.56–0.66)] and always wearing face coverings [predicted probability range 0.71 (95% CI 0.66–0.75) – 0.80 (95% CI 0.76–0.84) across survey periods]. There were significant cross-occupational trends towards reduced mitigations during periods of less stringent national restrictions. The majority of participants across occupations (55–88%) agreed that most mitigations were reasonable and worthwhile even after the relaxation of national restrictions; agreement was lower for physical distancing (39–44%).

**Conclusions:**

While usage of work-related mitigations appeared to vary alongside stringency of national restrictions, agreement that most mitigations were reasonable and worthwhile remained substantial. Further investigation into the factors underlying between-occupational differences could assist pandemic planning and prevention of workplace COVID-19 transmission.

A diverse range of mitigation methods have been employed during the COVID-19 pandemic to reduce SARS-CoV-2 transmission in workplaces. These have included workplace closures, behavioral and environmental measures to reduce transmission (eg, testing programmes and requirements to isolate from work if infectious, requirements or recommendations to wear face coverings, spatial reconfiguration to promote social distancing, ventilation), and promotion of COVID-19 vaccination by employers. Effective implementation of pandemic-related mitigation measures is likely to vary substantially by occupation due to variability in work environments, job roles and work cultures, and according to time-varying legislation and guidance at the national, sectoral, and workplace levels. Occupational differences in SARS-CoV-2 infection risk have been observed across the pandemic (1–4), and continue to present concerns in terms of workforce disruption and long-term disability even with the availability of safe and effective COVID-19 vaccines. Occupational differences in mitigation measures are likely to interact with workplace exposure to shape infection risk and are consequently an important area for cross-sector investigation. Additionally, understanding how mitigation methods have been implemented across occupations is important to inform effective and economically viable planning for future public health threats. However, multi-occupation investigation into mitigation methods is currently limited.

Modelling, simulation and observational studies of workplace COVID-19 prevention and control strategies indicate that layered packages of mitigation methods – including gradual return to in-person working, asymptomatic testing, reduction of contact, and using personal protective equipment (PPE) – appear effective and more likely to reduce worker test positivity compared to single measures (5–8). However, studies thus far have tended to focus on single workplaces, and many sectors outside of health and social care settings are underrepresented in the current literature (6). The effectiveness and feasibility of different packages of mitigations is likely to differ substantially across occupational sectors and roles, and multi-occupation observational studies are consequently warranted. In the UK – the regional focus of this study – this need is underscored by trade union reports indicating infrequent or inconsistent implementation of pandemic-related health and safety measures in a variety of workplaces during the first year of the pandemic (9, 10). Empirical investigation into the implementation of work-related mitigations across a range of occupational groups would provide both potential insight into occupational differences in infection risk and possible areas for intervention as well as evidence to plan for future public health emergencies.

This study aimed to investigate between-occupation and time-related differences in the implementation of workplace mitigation methods in England and Wales during key periods of pandemic-related national legislation between late December 2020 (third national lockdown in both nations) and late February 2022 (after relaxation of most pandemic restrictions). The specific objectives were to investigate: (i) how implementation and uptake of key work-related mitigations varied by occupation and, where relevant, by pandemic phase; and (ii) which COVID-19 mitigations methods participants perceived as reasonable and worthwhile in different occupations during the third national lockdown (late December 2020 – March 2021) and during a period of relaxed restrictions in February 2022.

## Methods

### Participants

Participants were an adult sub-cohort of the Virus Watch longitudinal cohort study (11), a prospective community cohort of 58 692 participants across England and Wales who were recruited between 1 June 2020 and 12 February 2022. The Virus Watch study recruited whole households using social media, SMS, and personalized postal recruitment supported by general practices, with the following inclusion criteria: ordinarily resident in England or Wales, household between 1–6 people (due to limitations on survey infrastructure), internet and email access, and ≥1 household member able to complete surveys in English. Further details of the main Virus Watch cohort and recruitment are provided in the study protocol (11). Participants from the main cohort were included in the present study if they met the following further criteria: an adult ≥16 years, who responded to a survey sent on 22 February 2022 regarding mitigation methods in the workplace, who was employed or self-employed and not on full-time furlough during at least part of the survey period, and who reported a classifiable (see Exposure section), consistent occupation throughout the survey period (when employed or self-employed).

### Exposure

Participants’ provided their main occupation as free text during registration with the Virus Watch cohort and at the beginning of the survey underlying this study (sent in February 2022). We used responses to the February 2022 survey as a preferred source due to direct coverage of the survey period. Responses from the baseline survey were used where participants reported being employed or self-employed but did not provide a classifiable occupation (N=586). As the survey was displayed only to participants who indicated a consistent occupation throughout periods of employment covered by the survey (due to limitations with the survey infrastructure and complexity), we assumed that the baseline survey was likely to be representative and included these participants to strengthen sample size.

To classify occupation, we assigned UK Standard Occupational Classification (SOC) 2020 (12) codes using semi-automatic processing in Cascot Version 5.6.3. If participants reported multiple occupations, the first listed occupation was used. We then used SOC codes to classify participants into the following eleven occupational groups, which aimed to reflect workplace environment while retaining the overall structure of SOC-defined skill groupings where possible: healthcare occupations; teaching, education and childcare occupations; social care and community protective services; leisure and personal service occupations; indoor trade, process and plant occupations; administrative and secretarial occupations; sales and customer service occupations; transport and mobile machine operatives; managers, directors, and senior officials; other professional and associate occupations (broadly non-frontline, office-based professional occupations); and outdoor trade occupations.

The most prevalent SOC-2020-defined occupations for participants in this study are reported in the supplementary material (www.sjweh.fi/article/4092), table S1. Analyses could not be further disaggregated by specific occupations due to sample size limitations.

### Outcomes

All outcomes were derived from responses to a one-off survey about work-related mitigations sent on 22 February 2022 as part of the Virus Watch monthly survey, which also included other questionnaires about household composition and health that were unrelated to this analysis. All cohort participants over 16 years of age were prompted to complete the first section of the work-related mitigations survey, which concerned their employment status during the survey period (January 2021–February 2022), their occupation if employed or self-employed, and whether their occupation was consistent across the survey period. Participants who were employed or self-employed during at least part of the survey period were then asked if they attended their workplace in-person during these periods. Subsequent workplace-related items were only displayed to participants who attended in-person during a given period due to the nested survey structure (supplementary figure S1). Questions aimed to cover key aspects of work-related transmission risk and associated mitigations based on contemporary understanding of transmission pathways of SARS-CoV-2 (13–15) and UK governmental sources regarding COVID-19 legislation and recommendations applicable to workplaces (16, 17).The full Virus Watch Work-Related Mitigations Survey (February 2022 is available in the supplementary material.

The first section of the survey comprised items regarding implementation and usage of key COVID-related mitigation methods in the workplace. Items addressed key mitigation methods including social distancing, ventilation, usage of face coverings, usage of lateral flow tests (LFT), surface and hand hygiene, and promotion of COVID-19 vaccination. Items applicable across multiple periods of the pandemic and liable to substantially change were asked separately for the following periods of restrictions: late December 2020–March 2021 (third national lockdown in England and Wales), July–December 2021 (most restrictions relaxed during this period in both nations), late December 2021–January 2022 (Omicron/Phase 2 restrictions in both nations), or current survey period (most restrictions relaxed in both nations). The survey was limited to the period between late December 2020 to February 2022 to balance recall bias with collecting information across key periods of national legislation. Some items – particularly those relevant to risk-related workplace features – were adapted from previous sources including the COVID-19 Job Exposure Matrix: a six-dimension measure classifying occupational risk of SARS-CoV-2 transmission based on a range of workplace features (18), other Virus Watch surveys, and items about the Flu Watch prospective cohort study (19). Permission to use or adapt items was sought where required. Supplementary table S2 reports the source from which each item was adapted. Items measured across multiple periods were displayed only for periods when the participant reported being employed or self-employed, and items relating to the workplace environment were displayed only for periods with in-person attendance. The questionnaire structure is detailed further in supplementary figure S1.

In the second section of the survey, participants rated how reasonable and worthwhile they believed key mitigation methods in their workplace to be during the third national lockdown (most stringent period of restrictions covered by the survey) and the current phase of the pandemic at the time of the survey (late February 2022 after relaxation of most pandemic-related restrictions). Items were rated on a five-point Likert-type scale: strongly disagree (not at all reasonable or worthwhile) to strongly agree (very reasonable and worthwhile), with the additional potential response "not possible/relevant in my job".

### Covariates

Where required (see Statistical Analyses section), models were adjusted for the following covariates: age (<30, 30–39, 40–49, 50–59, ≥60 years), sex at birth, employment status (working <20, 20–35, >35 hours per week), and clinical vulnerability status (vulnerable versus non-vulnerable, based on reporting of any medical condition classified by official UK sources to denote vulnerability to severe COVID-19) (20). Age and sex were derived from responses to the Virus Watch registration survey, employment status was drawn from the February 2022 survey, and clinical vulnerability was derived based on data sources detailed elsewhere (20). Employment status was entered as a time-varying covariate, as this was asked separately for each period (see Virus Watch Work-Related Mitigations Survey in supplementary material).

### Statistical analyses

Ordinal or binomial logistic regression was used to investigate between-occupational differences for all outcomes in the first section of the survey, comprising items related to workplace attendance, workspace sharing and social distancing, working environment and ventilation, hand and surface hygiene, usage and provision of face coverings, precautions during breaks and work-related social activities, workplace policy and provision of LFT, and promotion of COVID-19 vaccination. For items measured across multiple periods (including items related to including workplace attendance, workspace sharing and degree of social distancing, hand and surface hygiene, usage of face coverings, precautions during breaks and work-related social activities, and workplace policy regarding LFT), cluster-robust standard errors were used to account for within-individual clustering. Wald tests based on a cluster-robust estimate of the variance matrix were used to assess evidence of an interaction between occupational group and time. Based on the Wald tests, an interaction term was included in the final model for all outcomes pertaining to multiple periods (Wald P<0.001) excluding frequency of hand hygiene (P=0.09) and surface hygiene (P=0.17) and degree of precautions taken during breaks (P=0.30), which demonstrated main effects for occupation and time. Where identified, these interactions indicated that the frequency of the outcomes changed over time differentially by occupation.

Based on the VanderWeele principle of confounder selection (21) and adjustment sets for previous analyses of workplace attendance during the pandemic (22), the model for in-person workplace attendance was adjusted for age, sex, employment status, and clinical vulnerability. This model was not adjusted for vaccination status, as vaccination status was not assumed to alter general patterns of attendance across the broad survey periods. Additionally, changes in vaccination status occur on a discrete day and could not be linked with the broad time periods represented in the survey; full two-dose vaccination of the adult population occurred throughout the first survey period and three-dose vaccination was only introduced for the majority of the adult population during the final period (23). The effect of socio-demographic factors on other outcomes was presumed to occur via the impact of occupation, time and/or workplace attendance, and subsequent workplace-related items were only displayed for periods of in-person attendance due to the nested survey structure (supplementary figure S1). "Unsure" responses were dropped from relevant regression models to retain ordinal scales for most items. Complete case analysis was performed based on available responses for each question and missing data were limited for covariates (table 1); the number of respondents varied by question due to the nested structure of the items (supplementary figure S1). The total number of responses per item and "Unsure" (excluded) responses per item are reported in supplementary table S3.

**Table 1:** Demographic characteristics of participants [IMD=indices of multiple deprivation]

Characteristic		Current study participants (N=6279)		Workers in Virus Watch cohort (N=20 258)
		N (%)		N (%)
Occupation				
	Healthcare	584 (9.3)		1686 (8.3)
	Teaching, education and childcare	661 (11)		2297 (11)
	Social care and community protective Services	368 (5.9)		1117 (5.5)
	Leisure and personal service	304 (4.8)		1014 (5.0)
	Indoor trades, process and plant	411 (6.5)		1405 (6.9)
	Administrative and secretarial	880 (14)		2539 (13)
	Sales and customer service	295 (4.7)		1058 (5.2)
	Transport and mobile machine	139 (2.2)		479 (2.4)
	Managers, directors and senior officials	509 (8.1)		1653 (8.2)
	Other professional and associate	1935 (31)		6539 (32)
	Outdoor trades	193 (3.1)		471 (2.3)
Age (years)				
	<30	303 (4.8)		1796 (8.9)
	30–39	535 (8.5)		3457 (17)
	40–49	1112 (18)		4402 (22)
	50–59	2164 (34)		5728 (28)
	≥60	2165 (34)		4875 (24)
Sex				
	Female	3658 (58)		11 299 (55.8)
	Male	2603 (41)		8923 (44)
	Missing/other	18 (0.3)		36 (0.2)
Clinically vulnerability				
	Clinically vulnerable	2279 (36)		7031 (35)
	Not clinically vulnerable	4000 (64)		13 227 (65)
Ethnicity				
	White British	5401 (88)		16 411 (81)
	White Other	466 (7.6)		1855 (9.3)
	Mixed	71 (1.2)		346 (1.7)
	South Asian	86 (1.4)		906 (4.5)
	Other Asian	46 (0.8)		203 (1.0)
	Black	38 (0.6)		214 (1.1)
	Other ethnicity	25 (0.4)		118 (0.6)
	Missing	146 (2.3)		205 (1)
IMD quintile				
	1	546 (8.8)		2081 (10)
	2	973 (16)		3480 (17)
	3	1266 (20)		4089 (20)
	4	1611 (26)		4944 (24)
	5	1800 (29)		5390 (27)
	Missing	83 (1.3)		274 (1)
Region				
	East Midlands	600 (9.6)		1799 (8.9)
	East of England	1222 (19)		3889 (19)
	London	860 (14)		3565 (17)
	North East	254 (4.0)		882 (4.4)
	North West	617 (9.8)		2031 (10)
	South East	1261 (20)		3846 (19)
	South West	517 (8.2)		1388 (6.9)
	Wales	199 (3.2)		547 (2.7)
	West Midlands	354 (5.6)		1058 (5.2)
	Yorkshire and The Humber	312 (5.0)		979 (4.8)
	Missing	83 (1.3)		274 (1)

For the survey items pertaining to participants’ perceptions of mitigation methods in the workplace, we calculated response proportions stratified by occupational group and period. Descriptive analysis was performed as these items were intended to illustrate how workers’ perceptions varied across mitigation methods. Direct occupational comparison was not the objective of this analysis. This is in contrast to the first section of the survey, which was intended to investigate occupational differences in potentially risk-relevant features and mitigation methods over time.

## Results

Participants’ (N=6279) demographic features are reported in table 1, along with all workers with known occupation in the Virus Watch cohort to investigate potential response bias. Demographic features were similar between survey participants and the full cohort of workers, with some increased representation of older workers and those of a White British background amongst survey respondents. Participant selection illustrated in supplementary figure S2 and employment status over time in supplementary table S4. Of the 6660 participants who completed the survey, 6279 were eligible for inclusion in the present study due to reporting a consistent occupation (supplementary figure S2). The most common response for employment status across all survey periods was working >35 hours per week (47–48% of respondents across periods, supplementary table S4).

### Workplace sharing and social distancing

Across all periods, in-person workplace attendance was highest for tradespeople, transport, and leisure and personal service workers [predicted probability (PP) range 0.43 (95% confidence interval (CI) 0.37–0.50) to 0.58 (95% CI 0.52–0.64)] and lowest for other professional and associate occupations [PP 0.05 (95% CI 0.0–0.06) to 0.14 (95% CI 0.13-0.16)] (supplementary figures S3 and S4). Intensity of workspace sharing varied between occupations over time and was most intense for teaching, education, and childcare occupations and sales and customer service occupations; however, workspace sharing was common across occupations (Supplementary figure S5). Predicted probabilities for the workspace always being socially distanced were relatively low across all occupations (supplementary figure S6), even during the third national lockdown: PP range for this period 0.05 (95% CI 0.04–0.07) to 0.22 (95% CI 0.17–0.26). Healthcare workers and teaching, education and childcare workers persistently demonstrated the highest probabilities of reporting no social distancing at work [PP range 0.15 (95% CI 0.12–0.17) to 0.24 (95% CI 0.21–0.27)], with CI exceeding estimates for most other groups.

**Figure 1:**
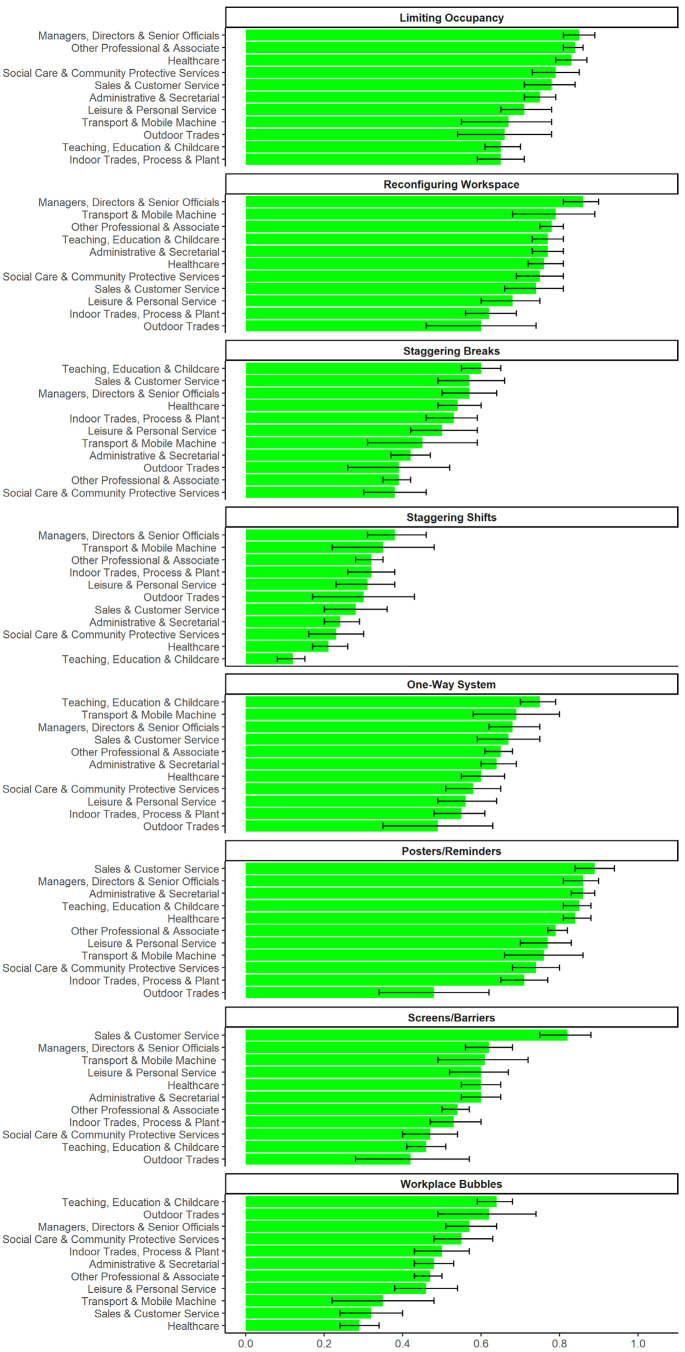
Strategies to reduce contact-related risk in the workplace: Predicted probabilities and 95% confidence intervals for reporting that workplace had ever used given strategy, by occupation. Note: responses collected for the whole survey period.

Strategies used in the workplace to promote social distancing varied by occupation (figure 1). All occupations had predicted probabilities around or >50% for reconfiguring the workspace [PP range 0.60 (95% CI 0.46–0.74) to 0.86 (95% CI 0.81–0.90)], limiting occupancy (PP range 0.65 (95% CI 0.59–0.71) to 0.85 (95% CI 0.81–0.89)], using one-way systems [PP range 0.49 (95% CI 0.35–0.63) to 0.75 (95% CI 0.70-0.79)], and using posters/reminders [PP range 0.48 (95% CI 0.34–0.62 to 0.89 (95% CI 0.84–0.94)]. Tradespeople tended to have lower probabilities than many groups across a range of methods (figure 1).

### Ventilation

Working environment by occupation is reported in supplementary figure S7. Except for outdoor tradespeople and transport occupations, the majority of participants across occupations reported working primarily indoors. Amongst participants who worked at least partly indoors, physical ventilation was the most commonly-reported method across groups (supplementary figure S8): PP range 0.64 (95% CI 0.56–0.73) to 0.93 (95% CI 0.85–1.00). Predicted probabilities for mechanical ventilation (supplementary figure S8) ranged from 0.25 (95% CI 0.21–0.29) to 0.60 (95% CI 0.56–0.63), and for air purifiers or filters PP ranged from 0.12 (95% CI 0.05–0.15) to 0.28 (95% CI 0.22–0.34). Teaching, education and childcare workers had the lowest likelihood of reporting these measures and managerial and other professional and associate occupations the highest.

### Hand and surface hygiene

Frequency of touching shared surfaces and objects is reported in supplementary figure S9. The probability of very frequently touching shared surfaces and objects was lower in outdoor trades [0.09 (95% CI 0.06–0.12)] and higher in healthcare, sales and customer service, leisure and personal service, and teaching, education and childcare occupations [PP range 0.39 (95% CI 0.35–0.44) to 0.46 (95% CI 0.41–0.50)] compared to all other groups.

**Figure 2:**
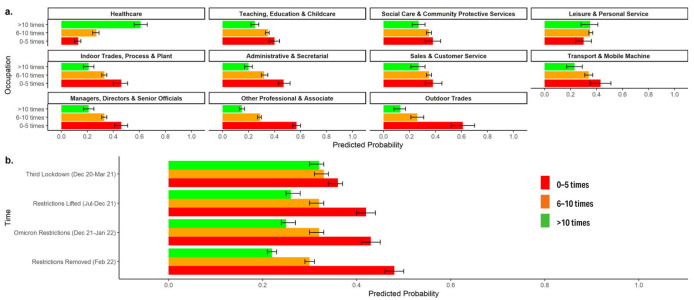
Frequency of handwashing at work: Predicted probabilities and 95% confidence intervals for frequency levels, by occupation (a) and over time (b). Note: responses collected separately for major periods of restrictions; interaction term (occupation× period) included based on Wald test.

Frequency of hand hygiene in the workplace varied substantially by occupation (figure 2a) and over time (figure 2b) with no interaction. Healthcare occupations had greater probability of reporting frequent (>10 times per workday) hand hygiene than other occupations [PP 0.61 (95% CI 0.56–0.66)]. Across all occupations, the probability of reporting infrequent hand hygiene (0–5 times per workday) increased between the third national lockdown [PP 0.36 (95% CI 0.33–0.37)] and subsequent periods [PP range 0.42 (95% CI 0.40–0.44) to 0.48 (95% CI 0.46–0.50)]; the probability of reporting frequent hand hygiene decreased correspondingly over time.

Similar occupational and time-based patterns were observed for frequency of surface hygiene (supplementary figures S10a and S10b).

### Face coverings

Healthcare workers were persistently the most likely group to self-report always using a face covering at work (supplementary figure S11): PP range 0.71 (95% CI 0.66–0.75) to 0.80 (95% CI 0.76–0.84). Social care, teaching, education and childcare, leisure and personal service, and sales occupations also had high initial reporting, though this dropped significantly by February 2022. Healthcare workers also had greater probability of reporting that other people on the worksite always wore face coverings compared to any other occupational group (supplementary figure S12). However, following cross-occupational statistical trends indicated by 95% CI (supplementary figure S12), this probability decreased over time [PP 0.68 (95% CI 0.64–0.73) for third national lockdown versus 0.48 (95% CI 0.43–0.52) for February 2022]. Workplaces were more likely to provide face coverings to healthcare workers [PP 0.96 (95% CI 0.94–0.98)] and other people attending healthcare settings [PP 0.90 (95% CI 0.87–0.93)] than any other occupational group (supplementary figure S13).

### Breaks and work-related social activities

Typical contact with others during breaks is reported in supplementary figure S14. Spending breaks indoors with other people was relatively common across occupations [PP range 0.21 (95% CI 0.13–0.29) to 0.53 (95% CI 0.48–0.57)]. Participants commonly reported that fewer pandemic-related precautions were taken during breaks (supplementary figure S15) compared to active work across occupations: PP range 0.39 (95% CI 0.35–0.44) to 0.54 (95% CI 0.46–0.61). For all groups, the probability of reporting fewer precautions during breaks increased over time, ranging from PP 0.30 (95% CI 0.28–0.32) during the third national lockdown to 0.59 (95% CI 0.57–0.61) in late February 2022. There was no interaction between occupation and time for this outcome.

Work-related social gatherings (supplementary figure S16) were relatively uncommon across all occupations during all periods [PP range for never occurring: 0.48 (95% CI 0.42–0.55) to 1.00 (95% CI 1.00–1.00)]. However, CI indicated a significant increase over time towards reporting social gatherings for most occupational groups except tradespeople, sales, and transport workers (supplementary figure S16).

### Lateral flow testing

**Figure 3:**
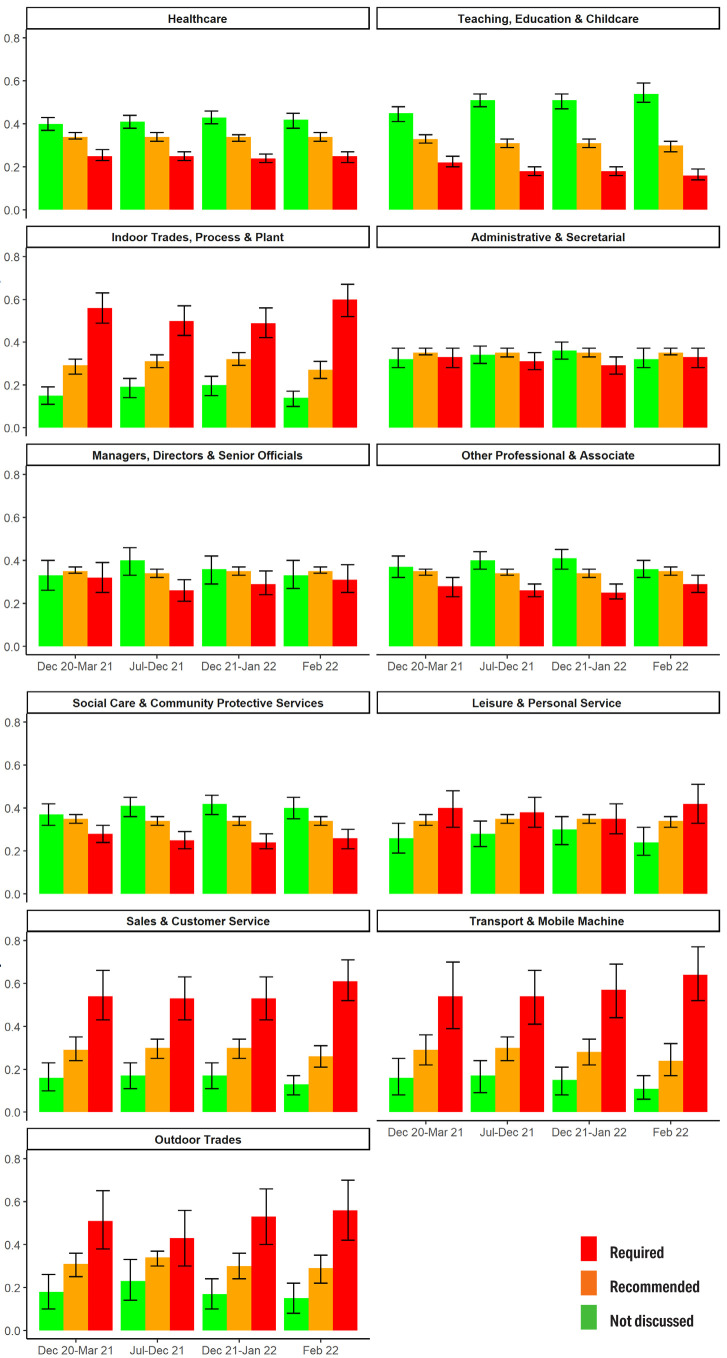
Lateral flow testing for workplace attendance: Predicted probabilities and 95% confidence intervals for workplace approach (testing required, recommended or not discussed), by occupation over time. Note: responses collected separately for major periods of restrictions; interaction term (occupation×period) included based on Wald test.

Occupational groups differed over time in their probability of regular LFT being required or recommended to attend work (figure 3). Teaching, education and childcare workers had the highest probability of requiring an LFT to attend the worksite across survey periods [PP range 0.45 (95% CI 0.41–0.48) to 0.54 (95% CI 0.50–0.59)]. Tradespeople, transport, and sales occupations had the highest probabilities of reporting no explicitly discussed workplace testing strategy [PP range for these groups across periods 0.51 (95% CI 0.38–0.65) to 0.64 (95% CI 0.52–0.77)].

The PP for workplace provision of LFT at any point during the study period (supplementary figure S17) ranged from 0.08 (95% CI 0.02–0.14) to 0.48 (95% CI 0.44–0.53) for on-site testing and from 0.23 (95% CI 0.15–0.31) to 0.83 (95% CI 0.80–0.87) for at-home test kits. Occupational patterns for LFT provision were similar to those for testing strategy.

### Workplace promotion of COVID-19 vaccination

**Figure 4:**
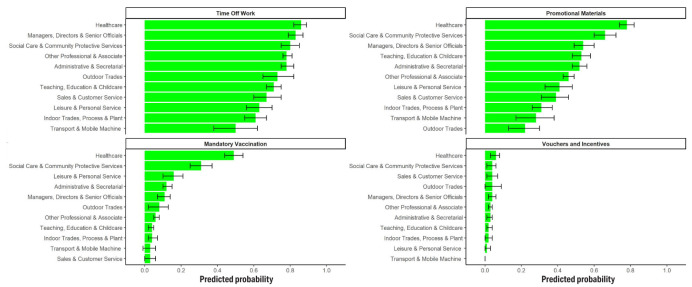
Strategies to promote COVID-19 vaccination: Predicted probabilities and 95% confidence intervals for reporting that workplace had ever used given strategy, by occupation. Note: responses collected for the whole survey period.

Workplace strategies to promote COVID-19 vaccination varied between occupations (figure 4). The most common method of promoting vaccination overall was providing time off work to attend vaccination appointments [PP range 0.50 (95% CI 0.38–0.62) to 0.86 (95% CI 0.82–0.89)], and use of promotional materials in the workplace [PP range 0.22 (95% CI 0.13–0.30) to 0.78 (95% CI 0.74–0.82)]. Use of vouchers was rare across all groups [PP range 0.00 (95% CI 0.00–0.00) to 0.06 (95% CI 0.03–0.08)], and mandatory vaccination was rare for most groups except health and social care workers [PP range 0.03 (95% CI 0.00–0.06) to 0.49 (95% CI 0.44–0.54)]. These two groups had the highest probabilities of reporting all strategies, and tradespeople and transport workers tended to have relatively low probabilities.

### Perception of work-related mitigation methods

Participants’ perceptions of key work-related mitigation methods are reported by occupation in supplementary figure S18 for the third national lockdown and supplementary figure S19 for late February 2022. Across all occupations during both periods, ≥50% of participants agreed or strongly agreed with each measure except physical distancing. Patterns of agreement were similar across occupations, and respondents agreed or strongly agreed, respectively, with the following measures in descending order: regular testing (88% during third national lockdown and 84% in February 2022), requiring face coverings for workers (88% and 84%), proof of vaccination for workers (86% and 74%), ventilation (83% and 62%), requiring face coverings for non-workers attending the worksite (83% and 62%), screens/barriers (79% and 60%), working from home (76% and 58%), surface cleaning (68% and 54%), proof of vaccination for non-workers attending the worksite (eg, customers, clients, patients) (62% and 55%), and physical distancing (44% and 39%). Outdoor tradespeople had relatively high levels of reporting that measures were not relevant or possible for their workplace across measures.

## Discussion

### Key findings and interpretation

This study found substantial between-occupational differences in risk-relevant workplace features and related mitigations. The relevance of work-related mitigations was illustrated by potential transmission risk, as workplaces tended to be shared and social distancing was often inconsistent, even during periods of stringent national restrictions. Occupational differences in the implementation of behavioral and environmental mitigations appeared to reflect differences in job roles, working environments, and related legislation and guidance. High usage across a range of mitigation methods in healthcare workers, for example, likely reflects pre- and peri-pandemic infection control protocols. Wide-ranging mitigations in some high-risk occupational groups (eg, healthcare) may have substantially mitigated workplace infection risk, potentially contributing to attenuated between-occupational differences in risk observed in longitudinal studies during this period compared to earlier pandemic periods (3, 4). This could not be directly investigated in this study.

In line with findings from earlier pandemic periods (22), there were significant cross-occupational trends towards more intense space sharing and fewer mitigations during periods of less intense national restrictions despite high levels of community transmission. However, the majority of respondents across all occupational groups (>50%) agreed or strongly agreed that most work-related mitigations – excluding physical distancing – were reasonable and worthwhile even after the relaxation of national restrictions. Workers may be more likely to adhere to public health interventions that they feel are reasonable and worthwhile (24). Further investigation into attitudinal determinants and impact of these perceptions, and continued communication between workers and those developing workplace guidelines could strengthen understanding and planning for future public health threats.

Given that risk-relevant workplace features were reported across occupations and time periods, reducing in-person workplace attendance was likely an important mitigation to reduce work-related transmission during the COVID-19 pandemic and remains relevant for future pandemic planning. However, remote working is not possible in many occupations, and workplace attendance and related mitigations were the focus of this study. Notably, we found that working remotely was the least well-supported mitigation measure by participants. Determinants of these perceptions were beyond the scope of this study, but the experience of remote working is likely to substantially depend on socio-economic position, housing conditions, and caring responsibilities amongst other factors (25, 26). Further investigation into attitudes to remote working and how to support remote working where possible is warranted to plan for future public health emergencies.

Several notable findings emerged regarding specific workplace features and/or mitigations. While physical ventilation was commonly reported across occupations, mechanical and filter methods – which tend to be more effective in many spaces (27) – were less prominent. Reporting was notably low for teaching, education and childcare occupations, and proactively scaling-up ventilation may increase sectoral resilience in the face of future public health threats given high-intensity contact in these essential occupations. Participants also commonly reported that people took fewer precautions during breaks compared to active work. Investigating effective support for maintaining protective measures during breaks is relevant for public health threats.

LFT was a flagship component of the government response in England and Wales and accrued high costs (28). Despite free availability for all individuals and businesses from April 2021, participants in non-target occupational groups (ie, outside of healthcare, social care, teaching and childcare) commonly reported no explicit guidance around testing and low workplace provision of tests. This was particularly likely for tradespeople and transport workers, despite persistently high in-person workplace attendance. These findings add to emerging evidence around sociodemographic inequalities in the implementation and uptake of mass testing (29). Clear communication and support are important, as recommendations and fear of income loss may reduce engagement with testing (30, 31). Participants’ personal usage of LFT devices was not investigated, but other behavioral surveys suggest low UK general population usage (31). Further investigation into why workplace-level guidance and test provision remained low across many sectors is recommended to identify policy-, employer-, and worker-related factors that may support uptake of future mass testing programmes.

Vaccination was another key element in the UK pandemic response, and national governments consequently encouraged workplaces to support their staff in taking up COVID-19 vaccines (32). Providing time off work (predicted probability range 50–86%) and use of promotional materials were relatively common (22-78%). However, occupations with the lowest level of workplace promotions – eg, trades and transport occupations – overlapped with those demonstrating relatively low vaccine uptake in previous studies (22, 33). A direct relationship cannot be inferred from this analysis, but investigation into effective workplace support for vaccination in these occupations may help to strengthen uptake in the event of future waves of COVID-19 or other vaccine-preventable outbreaks.

### Strengths and limitations

Strengths of these analyses included the large, multi-occupational cohort, and coverage of the third and fourth pandemic waves in England and Wales. These periods are currently underrepresented in the literature around occupation and COVID-19, and involved significant change in restrictions in England and Wales. These analyses address between-occupational differences in workplace mitigations across a range of occupations, which is an under-researched area. Responses were based on worker self-report, which – while potentially impacted by several biases discussed below – provides an important perspective regarding workplace hazards and protections and may be less prone to underreporting adverse workplace features to compared to official employer-level reporting (34).

Limitations include lack of population-representativeness, with participants comprising a higher proportion of older and clinically vulnerable workers. High representation of older and clinically vulnerable workers may have attenuated between-occupational differences due to these groups being prioritized for protective measures. Due to sample-size related constraints, it was not possible to further stratify results by age or vulnerability status. However, evidence of considerable between-occupational differences still emerged in this study. Self-selection of motivated participants – which is a common issue affecting community cohorts where random sampling is not possible (35) – may also have influenced responses. However, while self-selection may impact overall responding (eg, motivated participants may be more likely to engage in protective behaviors (36)), its impact on between-occupational differences is less clear and likely to be more limited.

The survey could only be delivered at a single timepoint and consequently a cross-sectional study design was required. Responses may consequently have been impacted by recall bias, with potentially lower accuracy of recall for early survey periods, although the direction of any consequent bias and its potential effects on between-occupational differences are unclear. Participants who experienced pandemic-related consequences, such as COVID-19 illness affecting themselves or their colleagues, may have differentially recalled risk-relevant features for relevant periods. Prospective longitudinal investigation was not possible due to constraints on survey content in the Virus Watch study. To mitigate recall bias, key periods of pandemic-related national restrictions were selected and the overall survey period was limited to approximately the previous year. Responses may also have been affected by social desirability bias – particularly where behaviors were subject to national guidance or employer-level mandates and may consequently have been subject to lower reporting. Generalizability to periods outside of those surveyed is unknown, though may be similar during periods of comparable restrictions. Stratification by nation (England versus Wales) was not possible due to sample size restrictions, but national guidance was similar during the survey periods. In Wales only, face masks were required in some public settings in February 2022 which may have impacted related items (37).

Due to burden- and delivery-related limitations, items were limited in detail and many items were measured using broad ordinal scales. The relationship between mitigations and infection risk could not be investigated due to data-related limitations.

### Concluding remarks

Risk-relevant workplace features and mitigation methods differed substantially between occupations and over time during the third and fourth pandemic waves in England and Wales. Between-occupational differences corresponded to occupational variation in workplace environments, job roles, and legislation and guidance. Across occupations, there were significant trends towards reduced mitigations during periods of less intense national restrictions on social mixing. However, participants appeared to have a high level of agreement with most mitigation methods in the workplace, even after the relaxation of most national-level restrictions. Further investigation into effective workplace support for flagship national mitigation initiatives, such as testing using LFTs and promotion of vaccination, may be warranted to inform future pandemic planning.

### Ethics approval

The Hampstead NHS Health Research Authority Ethics Committee approved the Virus Watch study (20/HRA/2320), which conformed to the ethical standards set out in the Declaration of Helsinki. All participants provided informed consent for all aspects of the study.

## Supplementary material

Supplementary material

## Data Availability

We aim to share aggregate data from this project on our website and via a “Findings so far” section on our website (ucl-virus-watch.net). We also share some individual record level data on the Office of National Statistics Secure Research Service. In sharing the data we will work within the principles set out in the UKRI Guidance on best practice in the management of research data. Access to use of the data whilst research is being conducted will be managed by the Chief Investigators (ACH and RWA) in accordance with the principles set out in the UKRI guidance on best practice in the management of research data. We will put analysis code on publicly available repositories to enable their reuse.
