# Supplementary material for: Between-occupation differences in work-related COVID-19 mitigation strategies over time: Analysis of the Virus Watch Cohort in England and Wales
Source: Scand J Work Environ Health. 2023 Jun 30;49(5):350–62. doi: 10.5271/sjweh.4092 (PMC10713985; doi:10.5271/sjweh.4092)
Supplement: Supplementary material [file SJWEH-49-350-S001.pdf]

Between-occupation differences in work-related COVID-19 mitigation strategies over time: Analysis of the Virus Watch Cohort in England and Wales[1]

by Sarah Beale, MSc,[2] Alexei Yavlinsky, PhD, Susan Hoskins, PhD, Vincent Nguyen, MSc, Thomas Byrne, MSc, Wing Lam Erica Fong, MSc, Jana Kovar, PhD, Martie Van Tongeren, PhD, Robert W Aldridge, PhD, Andrew Hayward, MD on behalf of the Virus Watch Collaborative

1. Supplementary Material
2. Correspondence to: Sarah Beale, Institute of Epidemiology and Health Care, University College London, London, UK, WC1E 7HB. [E-mail: sarah.beale.19@ucl.ac.uk]

Supplementary Table S1. UK Standard Occupational Classification 2020 (SOC-2020) Codes within Virus Watch Occupational Categories

Virus Watch Occupational Group	Included UK Standard Occupation Classification 2020 Unit Groups	Three Most Prevalent Occupations per Group* - UK Standard Occupation Classification 2020 Unit Groups
Healthcare Occupations (*n*=584 participants)	2211-2259, 3211-3219, 3240, 6131-6133	1. Other nursing professionals (26%, n=153)<br>2. Generalist medical practitioners (8.2%, n=48)<br>3. Community nurses (5.5%, n=32)
Teaching, Education & Childcare Occupations (*n*=661 participants)	1233, 2311-2329, 3231, 3232, 6111-6117, 9232	1. Higher education teaching professionals (16%, n=103)<br>2. Secondary education teaching professionals (12%, n=80)<br>3. Teaching assistant (12%, n=78
Social Care & Community Protective Services (*n*=368 participants)	1162, 1163, 1232, 2461-2469, 3221-3229, 3311-3319, 6134-6138, 6311-6312	1. Care workers and home carers (27%, n=98)<br>2. Welfare and housing associate professionals n.e.c. (12%, n=46)<br>3. Clergy (8.2%, n=30)

Leisure & Personal Service Occupations (*n*=304 participants)	1221-1225, 1252, 1253, 1256, 1257, 6121, 6129, 6211-6250, 9221-9229, 9231, 9261-9269	1. Cleaners and domestics (15%, n=46)<br>2. Kitchen and catering assistants (7.2%, n=22)<br>3. Waiters and waitresses (6.9%, n=21)
Indoor Trades, Process & Plant Occupations (*n*=411 participants)	5211-5250, 5315-5317, 5321-5323, 5411-5449, 8111-8149, 8160, 9131-9139, 9241-9259	1. Warehouse operatives (8.5%, n=35)<br>2. Metalworking production and maintenance fitters (8.3%, n=34)<br>3. Electricians and electrical fitters (7.5%, n=31)
Administrative & Secretarial Occupations (*n*=880 participants)	4111-4217, 9211, 9219, 9233	1. Other administrative occupations n.e.c. (25%, n=216)<br>2. Book-keepers, payroll managers, and wage clerks (11%, n=94)<br>3. Office managers (7%, n=62)
Sales & Customer Service Occupations (*n*=295 participants)	7111-7220	1. Sales and retail assistants (40%, n=117)<br>2. Customer service occupations n.e.c. (12%, n=36)<br>3. Retail cashiers and check-out operators (11%, n=31)
Transport & Mobile Machine Operatives (*n*=139 participants)	8211-8239	1. Large goods vehicle drivers (19%, n=26)<br>2. Road transport drivers n.e.c. (17%, n=23)<br>3. Delivery drivers and couriers (16%, n=22)
Managers, Directors & Senior Officials (*n*=509 participants)	1111-1161, 1171,1172, 1211, 1212, 1231, 1241-1243, 1251, 1254, 1255, 1258, 1259	1. Financial managers and directors (18%, n=93)<br>2. Managers and directors in retail and wholesale (10%, n=52)<br>3. Property, housing, and estate managers (8.6%, n=44)
Other Professionals & Associate Professionals (*n*=1935	2111-2162, 2411-2455, 2471-2494, 3111-3133, 3411-3582	1. Programmers and software development professionals (5.7%, n=111)

participants)		2. Management consultants and business analysts (4.1%, n=79)<br>3. Chartered and certified accountants (3.9%, n=75)
Outdoor Trade Occupations (*n*=193 participants)	5111-5119, 5311-5314, 5319, 5330, 8151-8159, 9111-9129	1. Gardeners and landscape gardeners (23%, n=44)<br>2. Construction and building trades n.e.c. (15%, n=28)<br>3. Farmers (12%, n=23)

Abbreviations: n.e.c. = not elsewhere classified; * Limited to three most prevalent occupations per category to prevent declarative disclosure and due to large number of occupations across sample (*n* =412). Occupations defined based on four-digit UK Standard Occupational Classification codes

Virus Watch Work-Related Mitigations Survey (February 2022)

Your Experiences at Work During the Pandemic

If you have been employed or self-employed during the pandemic, we would like to ask some questions about your work-related experiences.

We understand that people's working situations can vary considerably and can be difficult to capture in a questionnaire. There are no right or wrong answers so please just let us know about your situation.

1) **Which of the following descriptions best applies to your employment situation during the following periods?** If your employment status changed during any period, please select the description with the longest hours

Late December 2020 – March 2021 (third lockdown)	Not working (e.g. unemployed, retired) / On full-time furlough / Working up to 20 hours per week / Working 20-35 hours per week / Working more than 35 hours per week
July – December 2021 (restrictions lifted)	Not working (e.g. unemployed, retired) / On full-time furlough / Working up to 20 hours per week / Working 20-35 hours per week / Working more than 35 hours per week
Late December 2021 – January 2022 (Omicron/Phase 2 restrictions)	Not working (e.g. unemployed, retired) / On full-time furlough / Working up to 20 hours per week / Working 20-35 hours per week / Working more than 35 hours per week
Current	Not working (e.g. unemployed, retired) / On full-time furlough / Working up to 20 hours per week / Working 20-35 hours per week / Working more than 35 hours per week

2) *[If working full time/ part time at any point]* **Were you working in the same job or similar jobs/roles throughout?** Yes/ No

3) [If yes]: **What is/was your job? If you work(ed) in multiple jobs at the same time, please provide answers for the position that you would consider your 'main' job.** [Text]

4) *[If working full time/ part time at any point]*: **During the following period(s), how many days did you work <u>outside your home</u> in an average week?** Please round down to the nearest day (e.g. if you worked two and a half days, choose two)

Late December 2020 – March 2021 (third lockdown)	None / Half a day / 1 day / 2 days / 3 days / 4 days/ 5 or more days
July – December 2021 (restrictions lifted)	None / Half a day / 1 day / 2 days / 3 days / 4 days/ 5 or more days
Late December 2021 – January 2022 (Omicron/Phase 2 restrictions)	None / Half a day / 1 day / 2 days / 3 days / 4 days/ 5 or more days
Current	None / Half a day / 1 day / 2 days / 3 days / 4 days/ 5 or more days

5) *[If attended workplace at any point]* **Please select the description of your main working environment that best applies:** Working (mostly) outside/ Working partly inside / Working (mostly) inside

6) *[If works indoors at least sometimes]:* **Does your workplace regularly use any of the following ventilation methods? Please select all that apply:**

Opening doors and/or windows when weather allows	Yes/ No / Don't know/ NA
Mechanical ventilation system	Yes/ No / Don't know/ NA
Air purifiers/cleaners (e.g. HEPA filter)	Yes/ No / Don't know/ NA

7) *[For periods when attended workplace]* **During a typical workday, how many people did you share your workspace with across the whole day (even if socially distanced)?** Please provide your best estimate of the number of people you shared the workspace with across a typical day, including co-workers and customers/client/patients (if applicable).

Late December 2020 – March 2021 (third lockdown)	None / Fewer than 10 people / 10-30 people/ More than 30 people
July – December 2021 (restrictions lifted)	None / Fewer than 10 people / 10-30 people/ More than 30 people
Late December 2021 – January 2022 (Omicron/Phase 2 restrictions)	None / Fewer than 10 people / 10-30 people/ More than 30 people
Current	None / Fewer than 10 people / 10-30 people/ More than 30 people

8) *[If attended workplace at any point]* **Please indicate whether your workplace ever used any of the following measures to improve people's ability to social distance / to reduce contact:**

- Limiting the number of people on the premises: Yes / No / Don't know / NA
- Staggering the timing of breaks: Yes / No / Don't know / NA
- Staggering shifts: Yes / No / Don't know / NA
- Workplace bubbles (i.e. only working with a small, consistent group of colleagues): Yes / No / Don't know / NA
- One-way systems on the premises: Yes / No / Don't know / NA
- Reconfiguring the space (e.g. putting more space between tables or desks): : Yes / No / Don't know / NA
- Using posters, floor markings, or reminders to keep social distance: Yes / No / Don't know / NA
- Using screens or barriers: Yes / No / Don't know / NA

9) *[For periods when workspace was shared]* **Typically, to what degree could social distance (2m+) be maintained from other people in the same work area (e.g., colleagues, the public, patients)**

Late December 2020 – March 2021 (third lockdown)	Never/ Rarely/ Sometimes / Most of the Time/ Always
July – December 2021 (restrictions lifted)	Never/ Rarely/ Sometimes / Most of the Time/ Always
Late December 2021 – January 2022 (Omicron/Phase 2 restrictions)	Never/ Rarely/ Sometimes / Most of the Time/ Always
Current	Never/ Rarely/ Sometimes / Most of the Time/ Always

10) *[If attended workplace at any point]* **Typically at work, how often do you touch surfaces or other things that multiple people may also touch or use (e.g. tabletops, handrails, cash)?** Never/ Very infrequently/ Frequently/ Very frequently/Don't know

11) *[If surfaces ever shared]* **In general, how frequently were surfaces (e.g. tabletops, handrails) that are regularly touched by other people cleaned at your workplace?**

Late December 2020 – March 2021 (third lockdown)	Never/ Very infrequently/ Frequently/ Very frequently/Don't know
July – December 2021 (restrictions lifted)	Never/ Very infrequently/ Frequently/ Very frequently/Don't know
Late December 2021 – January 2022 (Omicron/Phase 2 restrictions)	Never/ Very infrequently/ Frequently/ Very frequently/Don't know
Current	Never/ Very infrequently/ Frequently/ Very frequently/Don't know

12) *[For periods when attended workplace]* **Typically, how frequently do you wash or sanitise your hands at work?**

Late December 2020 – March 2021 (third lockdown)	Never / 1-5 times a day / 6-10 times a day / more than 10 times a day
July – December 2021 (restrictions lifted)	Never / 1-5 times a day / 6-10 times a day / more than 10 times a day
Late December 2021 – January 2022 (Omicron/Phase 2 restrictions)	Never / 1-5 times a day / 6-10 times a day / more than 10 times a day
Current	Never / 1-5 times a day / 6-10 times a day / more than 10 times a day

13) *[For periods when workspace was shared]* **During a typical workday, how often were face coverings worn during the following periods?**

Late December 2020 – March 2021 (third lockdown)	**By you:** Never/ Rarely/ Sometimes / Most of the Time/ Always<br><br>**By others:** Never/ Rarely/ Sometimes / Most of the Time/ Always
July – December 2021 (restrictions lifted)	**By you:** Never/ Rarely/ Sometimes / Most of the Time/ Always<br><br>**By others:** Never/ Rarely/ Sometimes / Most of the Time/ Always
Late December 2021 – January 2022 (Omicron/Phase 2 restrictions)	**By you:** Never/ Rarely/ Sometimes / Most of the Time/ Always<br><br>**By others:** Never/ Rarely/ Sometimes / Most of the Time/ Always
Current	**By you:** Never/ Rarely/ Sometimes / Most of the Time/ Always<br><br>**By others:** Never/ Rarely/ Sometimes / Most of the Time/ Always

14) *[If workspace was shared]* **Did your workplace ever provide face coverings to reduce risk of transmission?**

For workers	No / Yes/ Don't know / NA
For customers/clients/patients	No / Yes/ Don't know / NA

15) *[If workspace was shared].***Typically, which of the following best describes how you spend breaktimes / lunchtime?** Alone / With others outdoors / With others indoors

16) *[For periods when workspace was shared]* **During the following periods, do you think that people in your workplace tended to be less likely to practice protective measures (e.g. social distancing, wearing face coverings) during rest periods or breaks?**

Late December 2020 – March 2021 (third lockdown)	Yes/ No/ Don't know/ NA
July – December 2021 (restrictions lifted)	Yes/ No/ Don't know/ NA
Late December 2021 – January 2022 (Omicron/Phase 2 restrictions)	Yes/ No/ Don't know/ NA
Current	Yes/ No/ Don't know/ NA

17) *[For periods when workspace was shared]* **Did your workplace organise social events outside of working hours or allow social events on work premises including food and/or drinks (i.e. parties)?**

Late December 2020 – March 2021 (third lockdown)	Never / Rarely / Sometimes / Frequently / NA
July – December 2021 (restrictions lifted)	Never / Rarely / Sometimes / Frequently / NA
Late December 2021 – January 2022 (Omicron/Phase 2 restrictions)	Never / Rarely / Sometimes / Frequently / NA
Current	Never / Rarely / Sometimes / Frequently / NA

18) *[For periods when attended workplace]* **Did your workplace require or recommend regular (e.g. twice weekly) COVID-19 lateral flow tests (swab tests where you get the result with 30 minutes) to attend work at any time during the following periods?**

Late December 2020 – March 2021 (third lockdown)	Required/Recommended/Not discussed/Don't know
July – December 2021 (restrictions lifted)	Required/Recommended/Not discussed/Don't know
Late December 2021 – January 2022 (Omicron/Phase 2 restrictions)	Required/Recommended/Not discussed/Don't know
Current	Required/Recommended/Not discussed/Don't know

19) *[If attended workplace at any point]* **Did your workplace ever provide lateral flow testing?**

On-site testing	Yes/ No/ Don't know
At-home test kits	Yes/ No/ Don't know

20) **How did your workplace promote COVID-19 vaccination, if at all. Please select all that apply:**

Allowed time off work to get vaccinated	Yes/ No / Don't know
Promotional materials (e.g. posters, staff emails)	Yes/ No / Don't know
Mandatory vaccination for work	Yes/ No / Don't know
Vouchers (or similar) for getting vaccinated	Yes/ No / Don't know
Other	Yes/ No / Don't know

21) **At <u>this time last year</u> (i.e., third national lockdown, prior to widespread vaccination of adults), how much do you agree that the following measures would have been reasonable and worthwhile to prevent COVID-19 transmission at your work?** We are interested in your opinions, even if the measures were not actually implemented at your work

	Strongly agree (very reasonable and worthwhile)	Agree	Neither agree nor disagree	Disagree	Strongly disagree (not at all reasonable or worthwhile)	Not possible/relevant in my job
Working from home when possible						
Regular Covid-19 tests for workers						
Proof of vaccination required for workers						
Proof of vaccination required for customers/clients						
Physical distancing (e.g., 2m or limiting number of customers)						
Workplace well-ventilated (e.g., opening doors/windows, ventilation system / air purification system)						
Face coverings required for workers						
Face coverings required for customers/clients/patients						
Using screens or barriers						
Regular cleaning of surfaces						

22) **At the <u>current stage</u> of the pandemic, how much do you agree that the following measures would be reasonable and worthwhile to prevent COVID-19 transmission at your work?** We are interested in your opinions, even if the measures were not actually implemented at your work

	Strongly agree (very reasonable and worthwhile)	Agree	Neither agree nor disagree	Disagree	Strongly disagree (not at all reasonable or worthwhile)	Not possible/relevant in my job
Working from home when possible						
Regular Covid-19 tests for workers						
Proof of vaccination required for workers						
Proof of vaccination required for customers/clients						
Physical distancing (e.g., 2m or limiting number of customers)						
Workplace well-ventilated (e.g., opening doors/windows, ventilation						

system/ air purification system)						
Face coverings required for workers						
Face coverings required for customers/clients/patients						
Using screens or barriers						
Regular cleaning of surfaces						

Supplementary Table S2. Focus and Source for Items in Work-Related Mitigations Survey

Item	Topic	Aim	Source
1	Employment status over time	Establishing eligibility	Virus Watch registration survey
2	Same job over time	Establishing eligibility	Created for survey
3	Job title	Establishing occupation (exposure)	Virus Watch registration survey
4	Days worked outside home	Screening for subsequent workplace related questions / potential exposure	Created for survey
5	Main working environment	Assess potential exposure (for ventilation item)	Adapted from COVID-19 JEM (1)
6	Ventilation methods	Assess mitigation - ventilation (aerosol transmission)	Created for survey
7	People in shared workspace	Assess potential exposure (for social distancing and other items)	Adapted from COVID-19 JEM
8	Methods to promote social distancing/reduce contact	Assess mitigation - social distancing (aerosol and droplet transmission)	Created for survey
9	Degree that social distance could be maintained	Assess mitigation - social distancing (aerosol and droplet transmission)	Adapted from COVID-19 JEM
10	Touching shared surfaces/objects	Assess potential exposure (for hygiene items)	Adapted from COVID-19 JEM
11	Frequency of surface/object cleaning	Assess mitigation – surface hygiene (indirect contact transmission)	Created for survey
12	Frequency of hand hygiene at work	Assess mitigation – hand hygiene (indirect/direct contact transmission)	Flu Watch Study (2)
13	Frequency of wearing face coverings	Assess mitigation – face coverings (primarily droplet transmission)	Adapted from COVID-19 JEM and Virus Watch contact survey (3)
14	Workplace provision of face coverings	Assess mitigation – face coverings (primarily droplet transmission)	Created for survey

15	How breaks/lunchtime spent	Assess potential exposure (for measures during breaks item)	Created for survey
16	Reduction in protective measures during breaks	Assess mitigation/exposure – stringency of measures during breaks (potentially all transmission pathways)	Created for survey
17	Work-related social events	Assess potential exposure – work-related social contact	Created for survey
18	Workplace LFT policy	Assess mitigation – case identification and isolation (all transmission pathways)	Created for survey
19	Workplace LFT provision	Assess mitigation – case identification and isolation (all transmission pathways)	Created for survey
20	Workplace COVID-19 vaccine promotion methods	Assess mitigation – reducing susceptibility esp. to severe outcomes	Created for survey
21	Perception of mitigation methods (third national lockdown)	Assess worker perceptions – period of high intensity of national measures	Created for survey
22	Perception of mitigation methods (February 2022)	Assess worker perceptions – period of low intensity of national measures	Created for survey

Abbreviations: COVID-19 JEM – COVID-19 Job Exposure Matrix; LFT – lateral flow test

References:

1 Oude Hengel KM, Burdorf A, Pronk A, Schlünssen V, Stokholm ZA, Kolstad HA, van Veldhoven K, Basinas I, van Tongeren M, Peters S. Exposure to a SARS-CoV-2 infection at work: development of an international job exposure matrix (COVID-19-JEM). Scand J Work Environ Health. 2022 Jan 1;48(1):61.

2 Beale S, Johnson AM, Zambon M, Flu Watch Group, Hayward AC, Fragaszy EB. Hand Hygiene Practices and the Risk of Human Coronavirus Infections in a UK Community Cohort. Wellcome Open Res. 2020;5:98.

3 Beale S, Hoskins S, Byrne T, Fong WLE, Fragaszy E, Geismar C, et al. Workplace contact patterns in England during the COVID-19 pandemic: Analysis of the Virus Watch prospective cohort study. Lancet Reg Health – Eur. 2022 May 1 [cited 2022 Oct 20];16.

Supplementary Figure S1. Schematic Diagram of Virus Watch Mitigations Survey: Items included in analyses

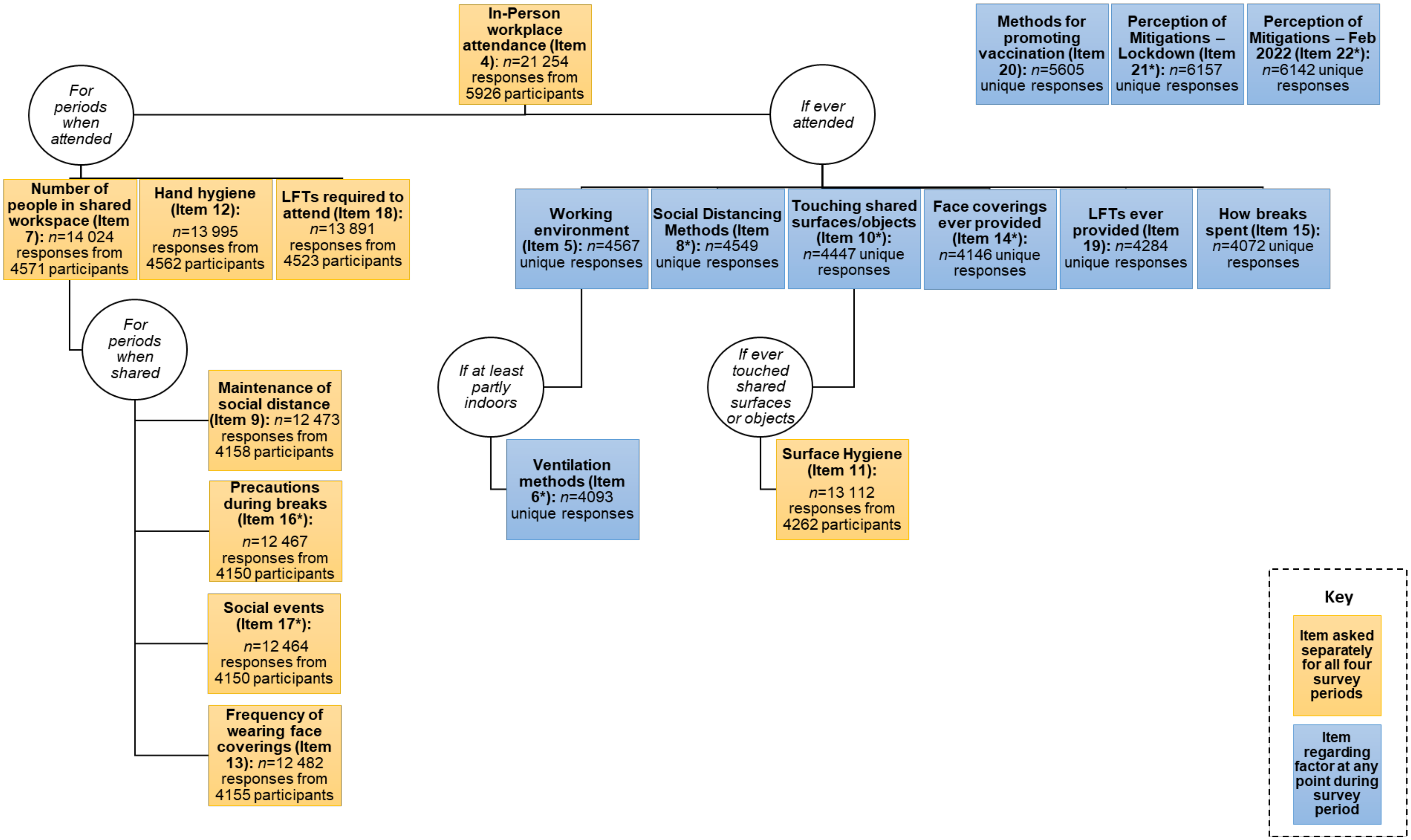

Note: Where item contained multiple sub-ratings, the overall maximum for the item is presented; * item contained 'Unsure/NA' responses, reported below

Supplementary Table S3. Excluded 'Unsure'/'NA' Responses by Item

Item	Item Timeframe	Unsure/NA Responses
6	Whole survey period	*n*=381 for physical ventilation<br>*n*=678 for mechanical ventilation<br>*n*=1153 for filter ventilation
8	Whole survey period	*n*= 571 for limiting occupations<br>*n*= 743 for reconfiguring workspace<br>*n*= 1374 for staggering shifts<br>*n*= 740 for one-way systems<br>*n*= 735 for screens/barriers<br>*n*= 918 for workplace bubbles<br>*n*= 618 for posters/reminders<br>*n*= 1229 for staggering breaks
10	Whole survey period	*n*=40 responses
11	Separate responses for all periods when workspace was shared	*n*=1748 responses for 649 participants
14	Whole survey period	*n*=316 for worker self-report<br>*n*=798 for other people on the worksite
16	Separate responses for all periods when workspace was shared	*n*=2571 responses for 951 participants
17	Separate responses for all periods when workspace was shared	*n*=1189 responses for 447 participants
18	Separate responses for all periods when workspace was shared	*n*=554 responses for 263 participants
19	Whole survey period	*n*=225 responses for on-site testing<br><br>*n*=183 responses for at-home test kits
20	Whole survey period	*n*= 970 for time off work<br>*n*= 845 for promotional material<br>*n*= 806 for vouchers<br>*n*= 665 for mandatory vaccination
21	Single timepoint (third national lockdown)	NA responses included in analyses due to different analytical method and aims – reported in main findings
22	Single timepoint (late February 2022)	NA responses included in analyses due to different analytical method and aims – reported in main findings

Note: Items not included in this table did not have 'Unsure' or 'NA' response options; NA=not applicable

Supplementary Figure S2. Flow Diagram of Participant Selection

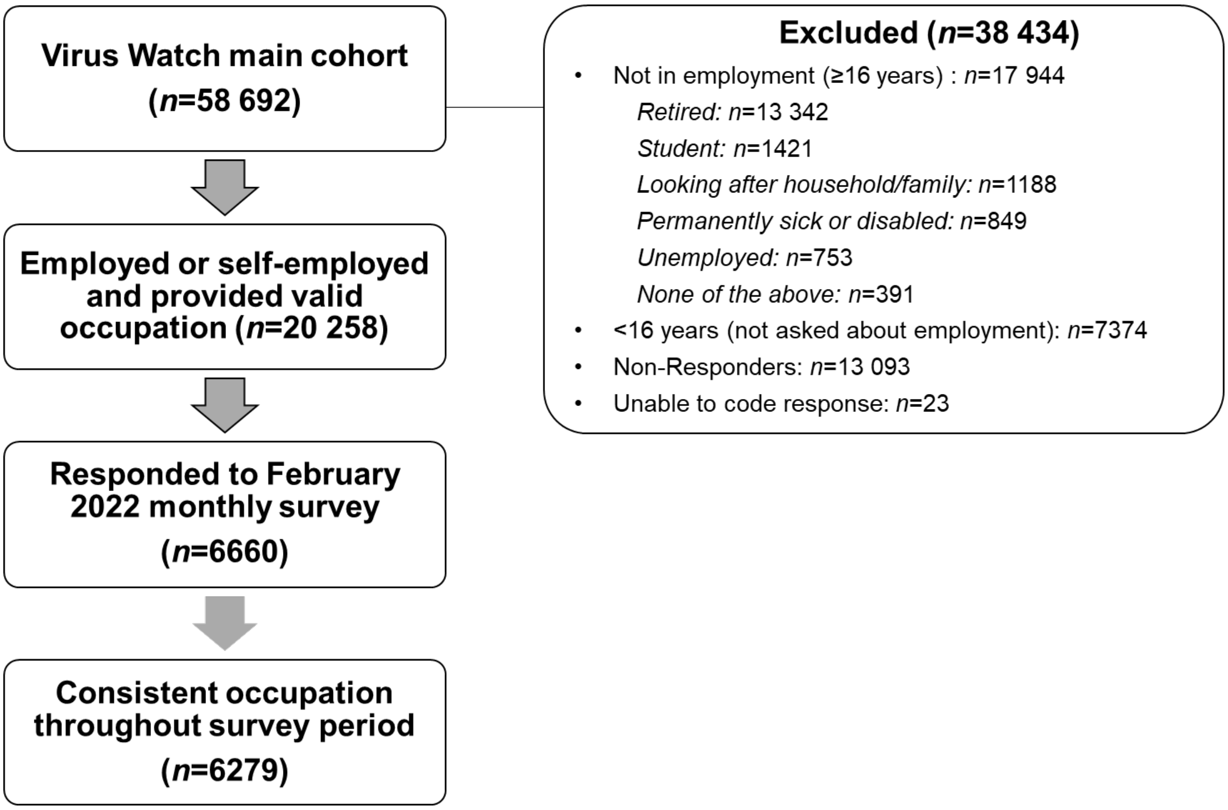

Supplementary Table S4. Employment Status Across Survey Periods

	Late December 2020 – March 2021 (N = 6225) n (%)	July – December 2021 (N = 6217) n (%)	Late December 2021 – January 2022 (N = 6206) n (%)	Late February 2022 (N = 6072) n (%)
Not working (e.g. unemployed, retired)	476 (7.6)	337 (5.4)	479 (7.7)	496 (8.2)
On full-time furlough	347 (5.6)	70 (1.1)	40 (0.6)	18 (0.3)
Working up to 20 hours per week	1,273 (20)	1,468 (24)	1,437 (23)	1,371 (23)
Working 20-35 hours per week	1,217 (20)	1,353 (22)	1,317 (21)	1,312 (22)
Working more than 35 hours per week	2,912 (47)	2,989 (48)	2,933 (47)	2,875 (47)

Supplementary Figure S3. Average Number of Days per Week Worked Outside the Home: Predicted probabilities and 95% confidence intervals by occupation over time (estimates adjusted for age, sex, employment status, and clinical vulnerability)

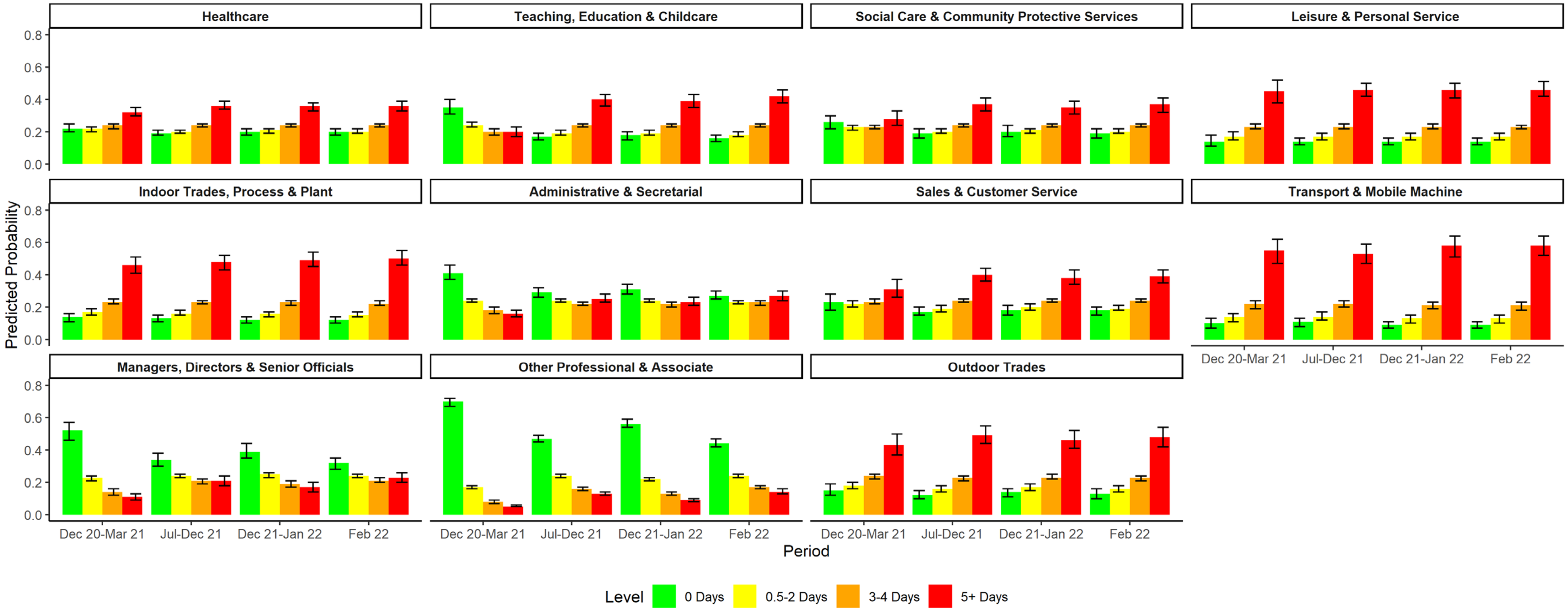

Note: Responses collected separately for major periods of restrictions; interaction term (occupation X period) included based on Wald test

Supplementary Figure S4. Average Number of Days per Week Worked Outside the Home: Unadjusted predicted probabilities and 95% confidence intervals by occupation over time

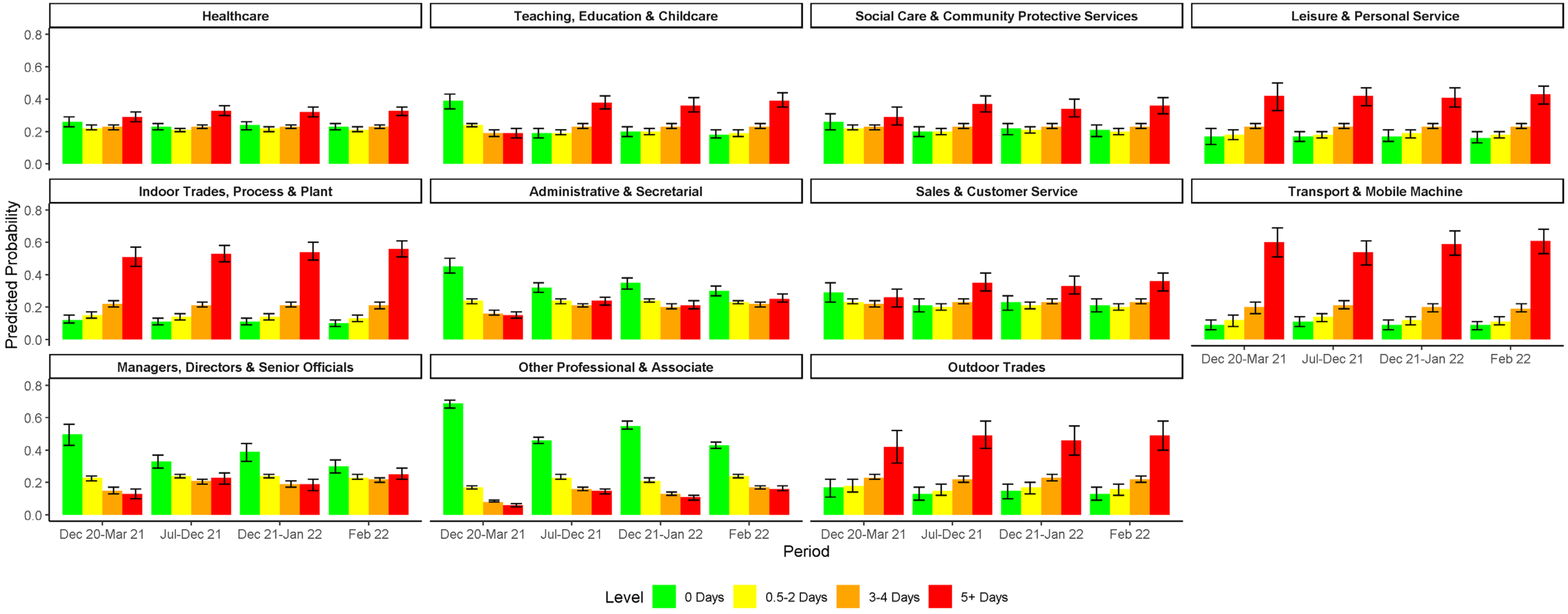

Note: Responses collected separately for major periods of restrictions; interaction term (occupation X period) included based on Wald test

Supplementary Figure S5. Number of People in Workspace across Day: Predicted probabilities and 95% confidence intervals by occupation over time

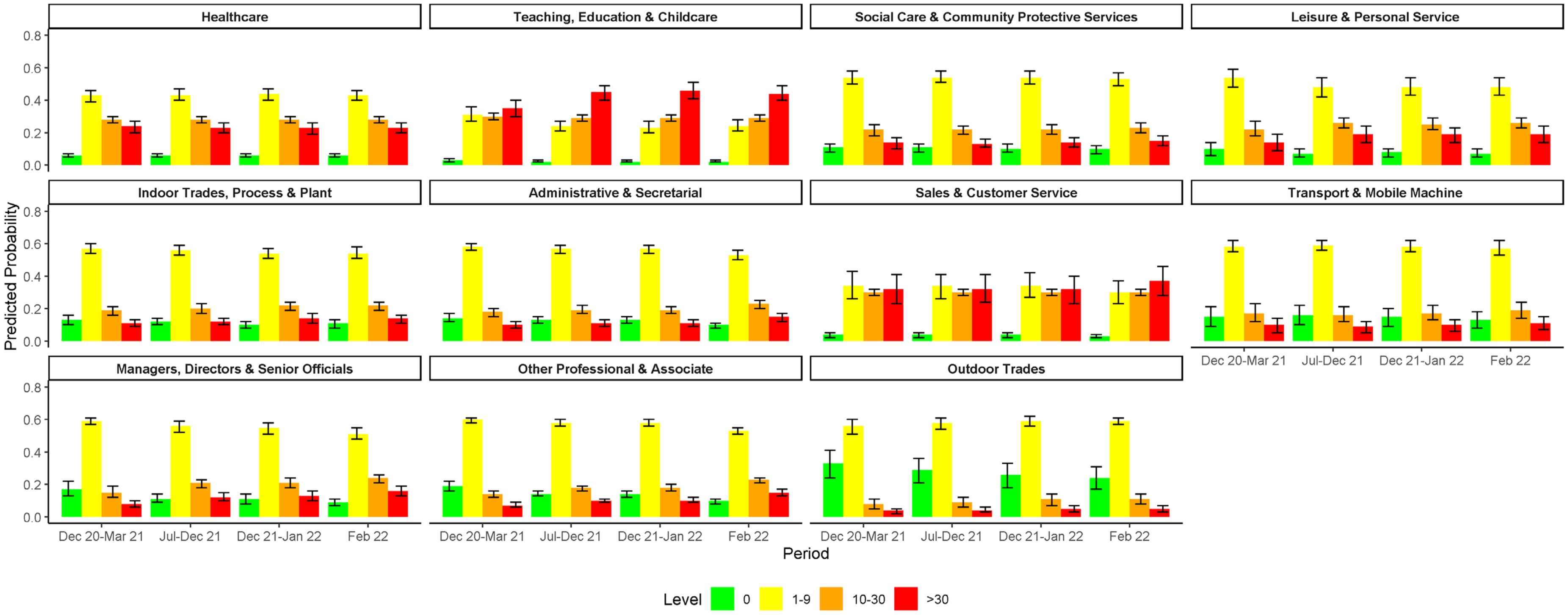

Note: Responses collected separately for major periods of restrictions; interaction term (occupation X period) included based on Wald test

Supplementary Figure S6. Reported Extent to Which Social Distance Could be Maintained at Work: Predicted Probabilities and 95% confidence intervals, by occupation over time

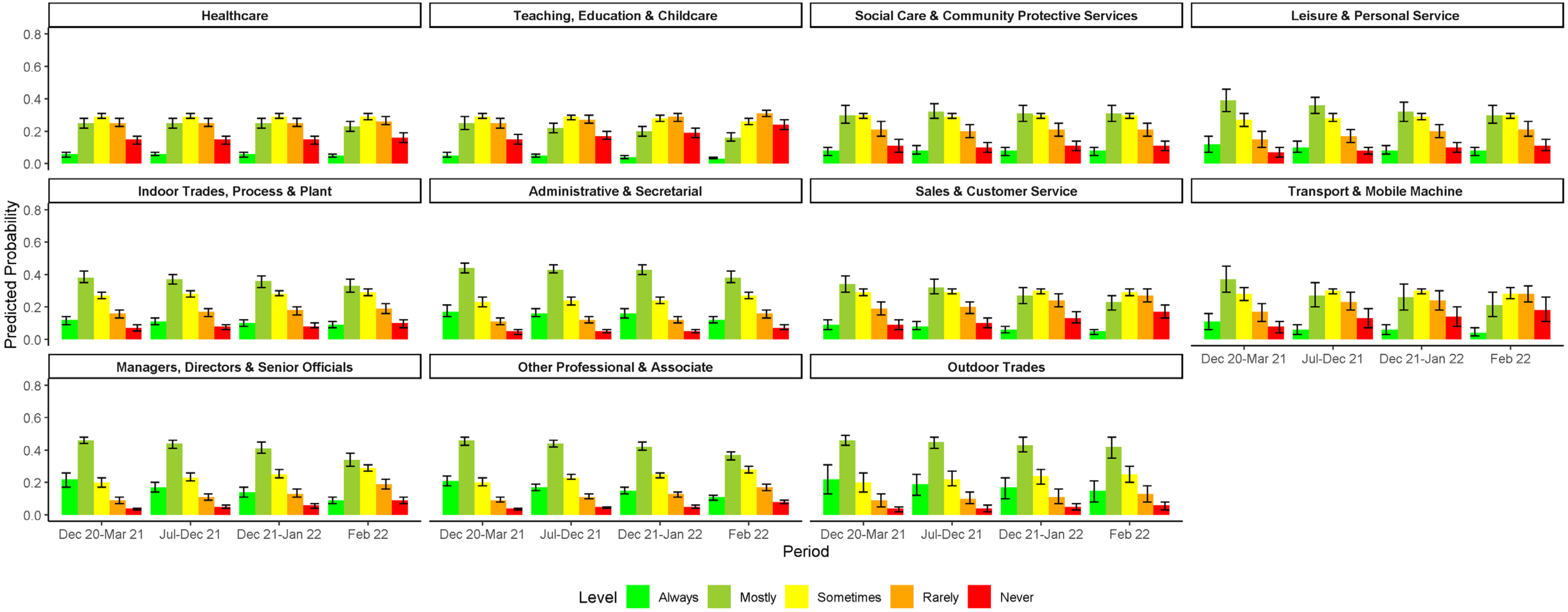

Note: Responses collected separately for major periods of restrictions; interaction term (occupation X period) included based on Wald test

Supplementary Figure S7. Primary Working Environment: Predicted probabilities and 95% confidence intervals for indoor and outdoor working by occupation

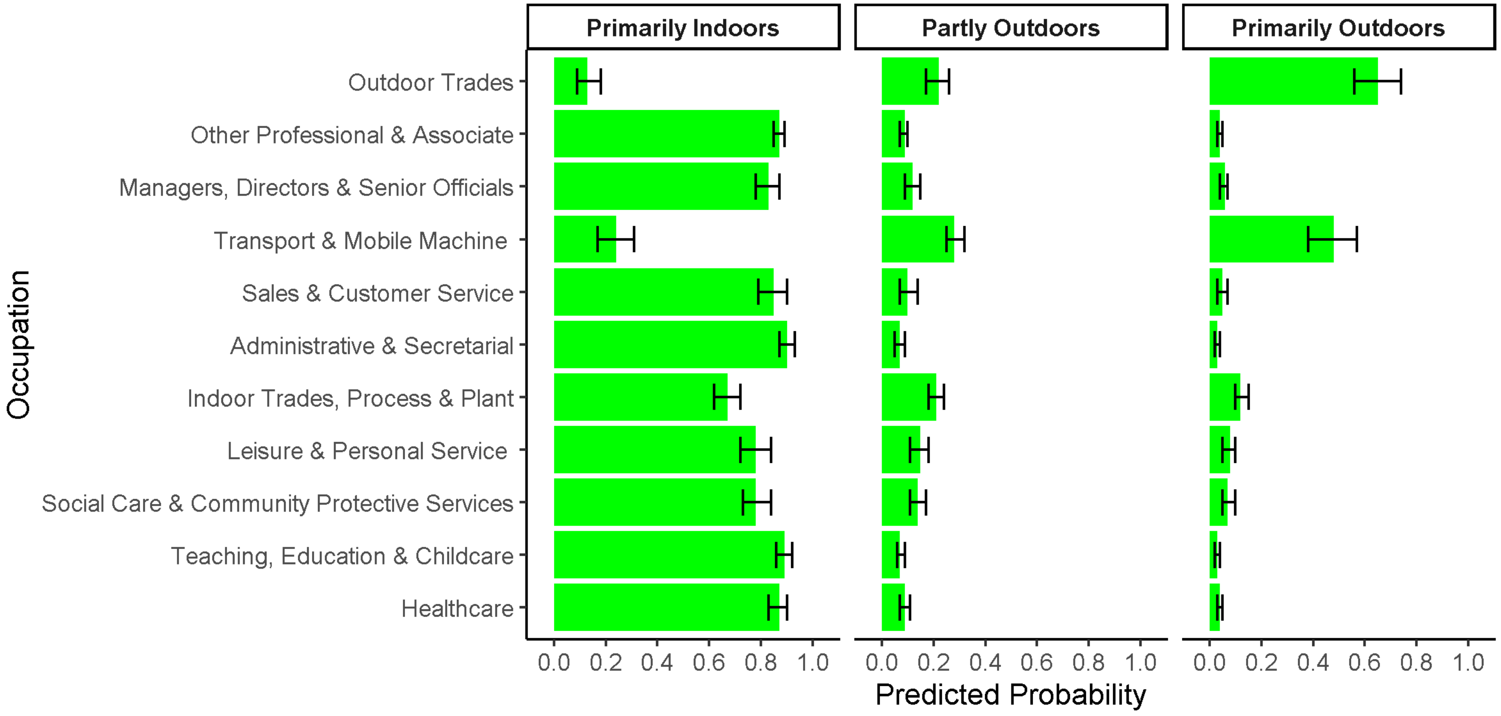

Note: Responses collected for whole survey period

Supplementary Figure S8. Ventilation Methods Used in the Workplace: Predicted probabilities and 95% confidence intervals for reporting that workplace had ever used given method, by occupation

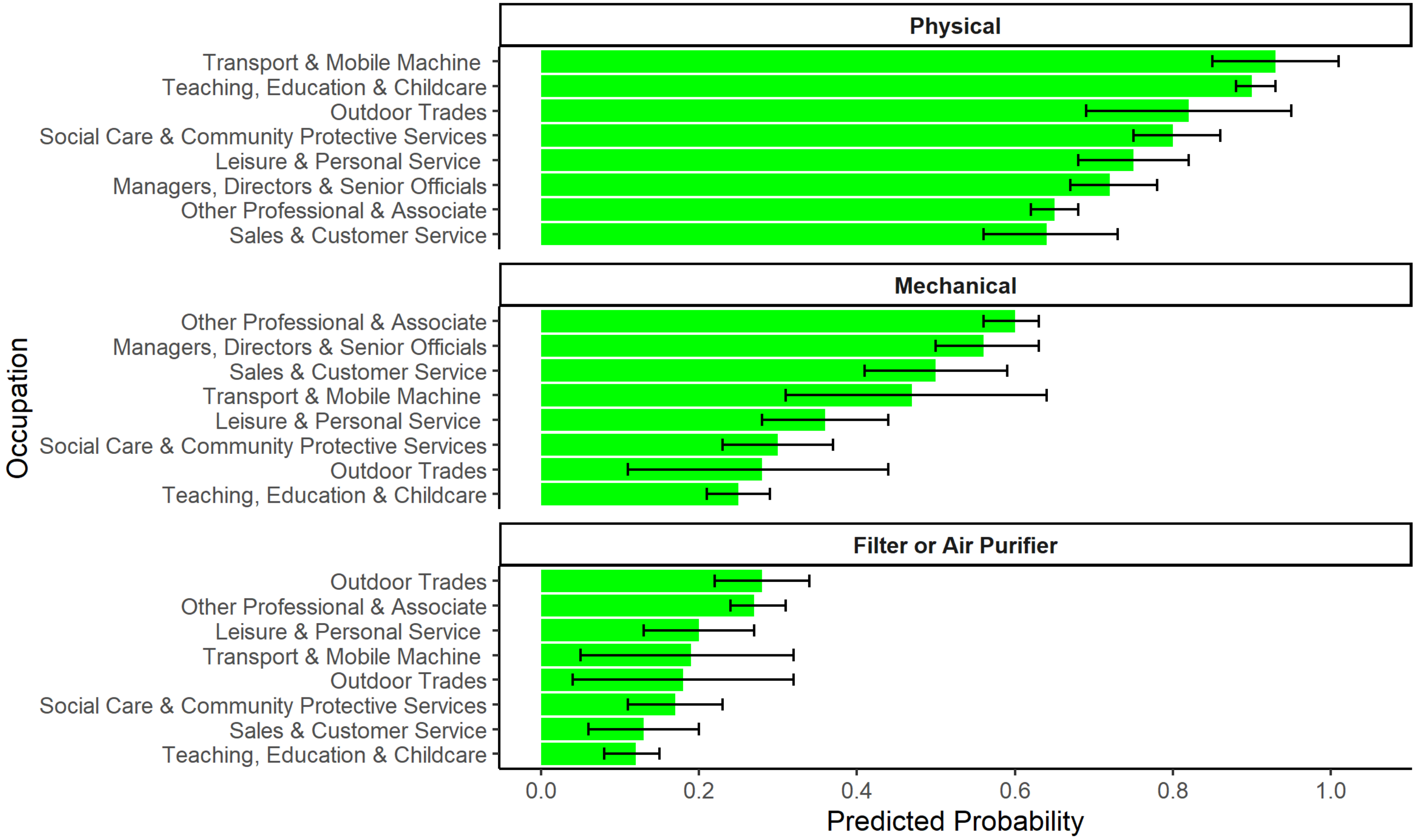

Note: Responses collected for whole survey period

Supplementary Figure S9. Frequency of Touching Shared Surfaces or Objects at Work: Predicted probabilities and 95% confidence intervals for frequency levels, by occupation

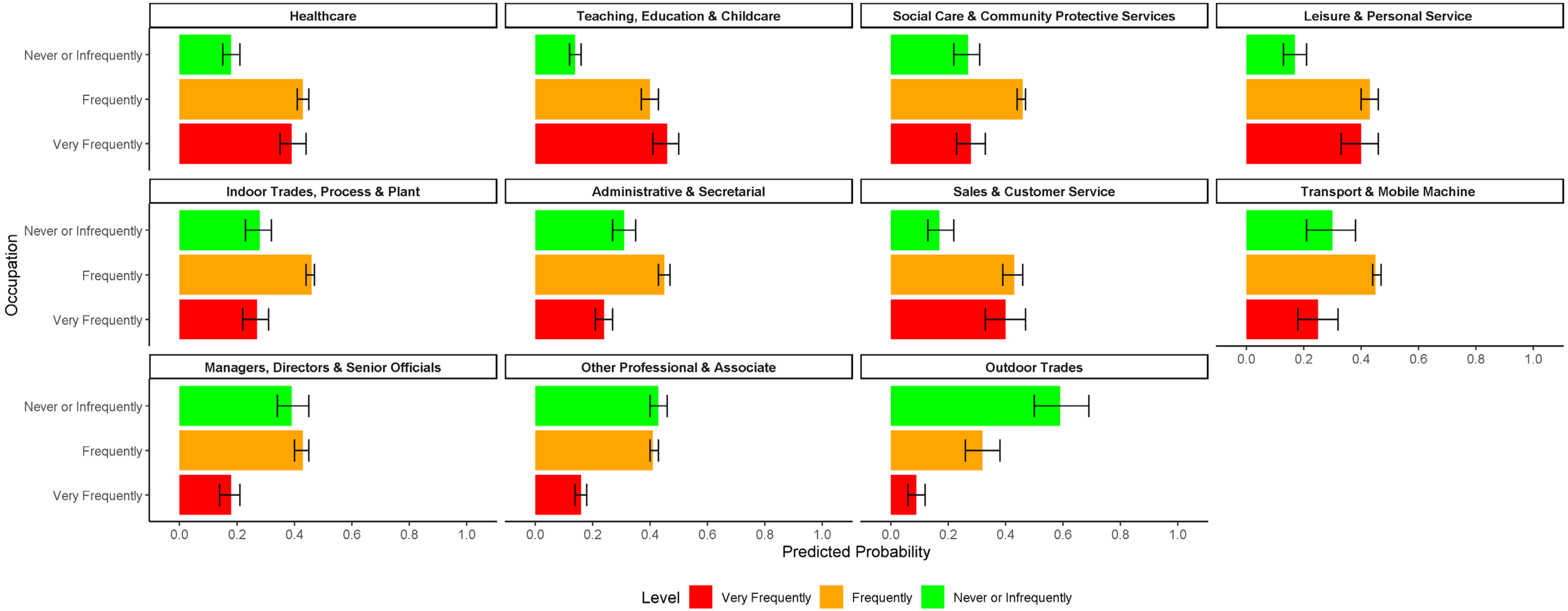

Note: Responses collected for whole survey period

Supplementary Figure S10. Frequency of Surface Hygiene at Work: Predicted probabilities and 95% confidence intervals for frequency levels, by occupation (a) and over time (b)

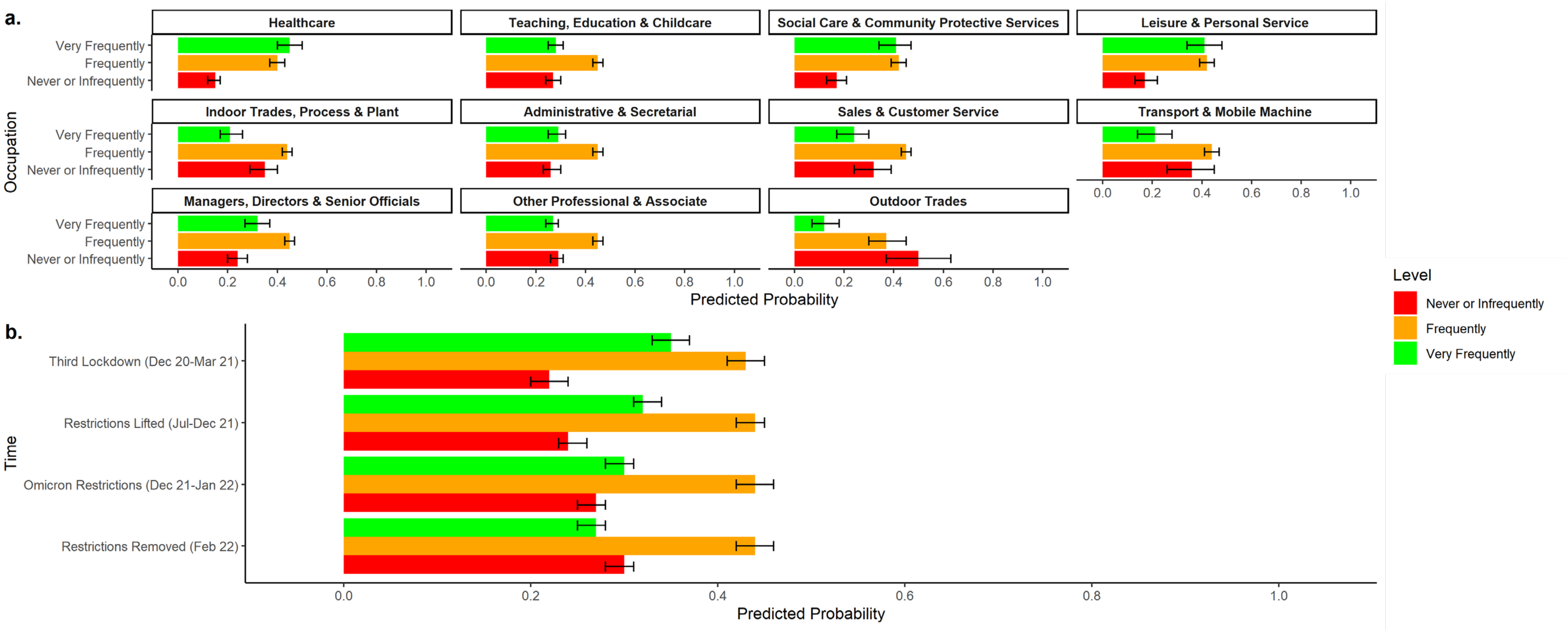

Note: Responses collected separately for major periods of restrictions; interaction term (occupation X period) not included based on Wald test

Supplementary Figure S11. Workers' Personal Usage of Face Coverings: Predicted probabilities and 95% confidence intervals for frequency levels, by occupation over time

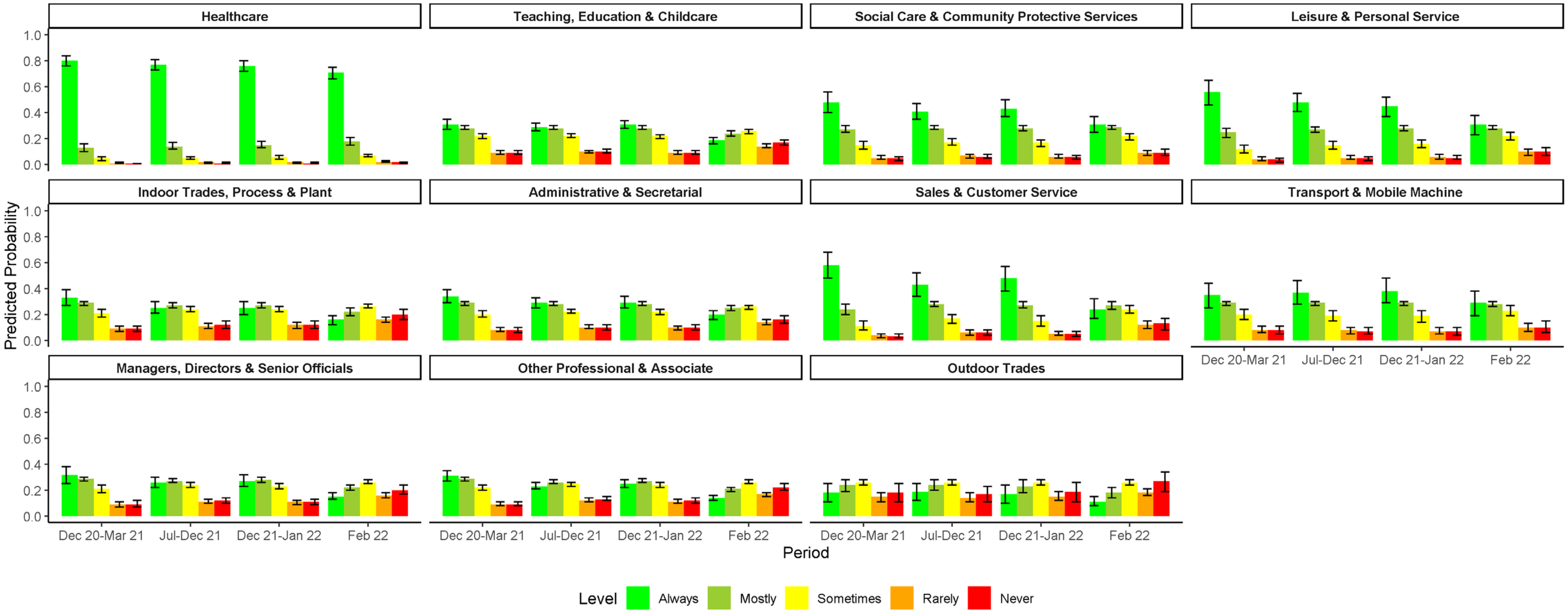

Note: Responses collected separately for major periods of restrictions; interaction term (occupation X period) included based on Wald test

Supplementary Figure S12. Usage of Face Coverings by Other People on the Worksite: Predicted probabilities and 95% confidence intervals for frequency levels, by occupation over time

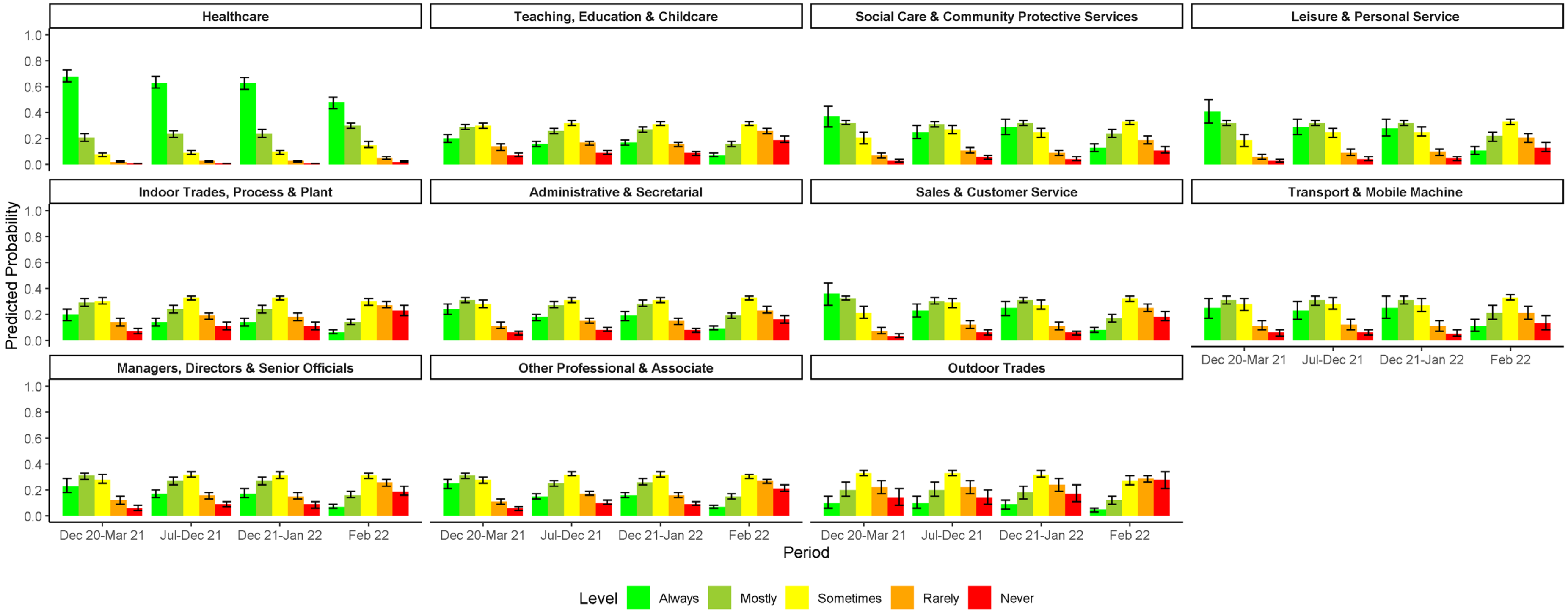

Note: Responses collected separately for major periods of restrictions; interaction term (occupation X period) included based on Wald test

Supplementary Figure S13. Workplace Provision of Face Coverings: Predicted probabilities and 95% confidence intervals that workplace had ever provided face coverings to given group, by occupation

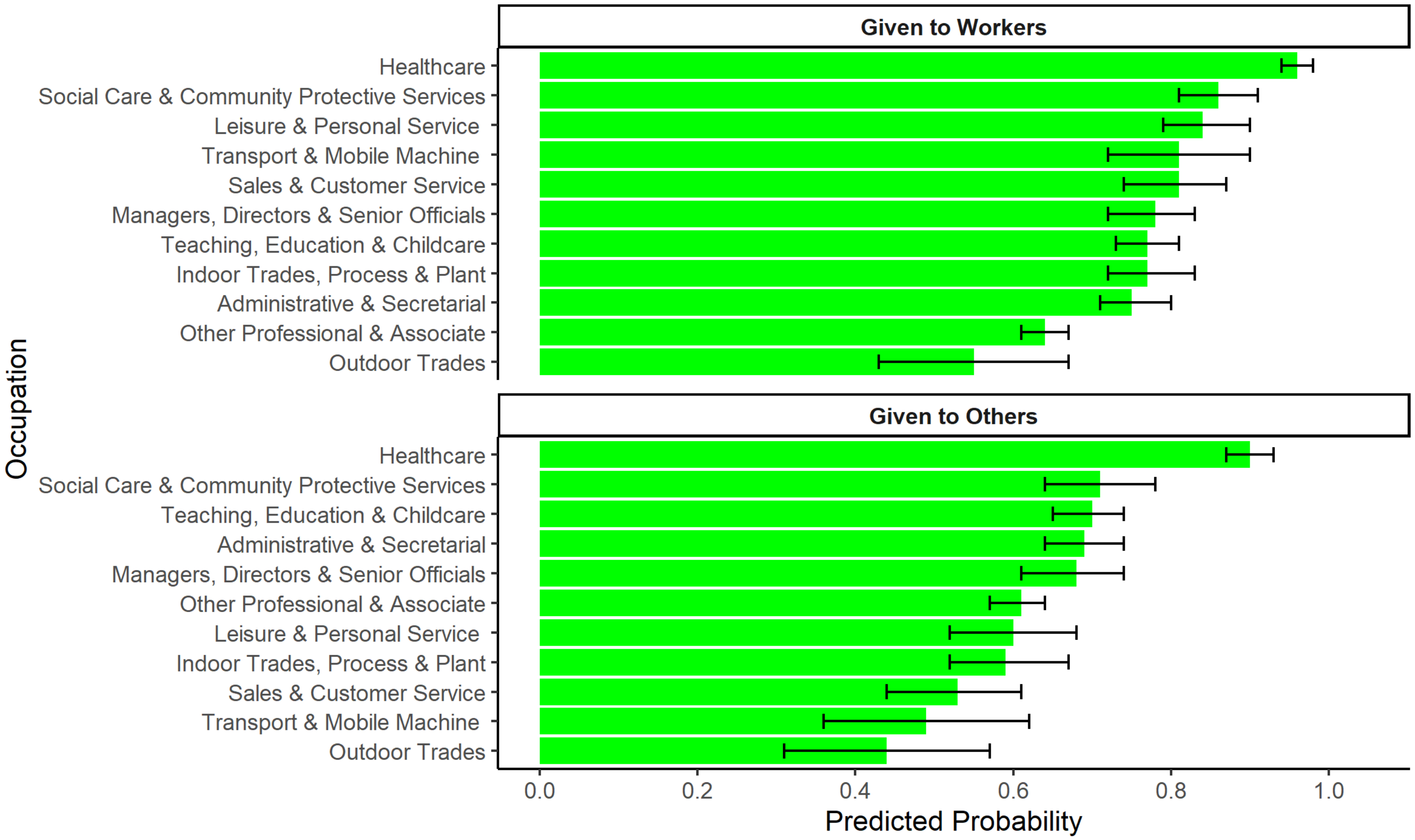

Note: Responses collected for whole survey period

Supplementary Figure S14. Typical Contact with Others during Breaks: Predicted probabilities and 95% confidence intervals by occupation

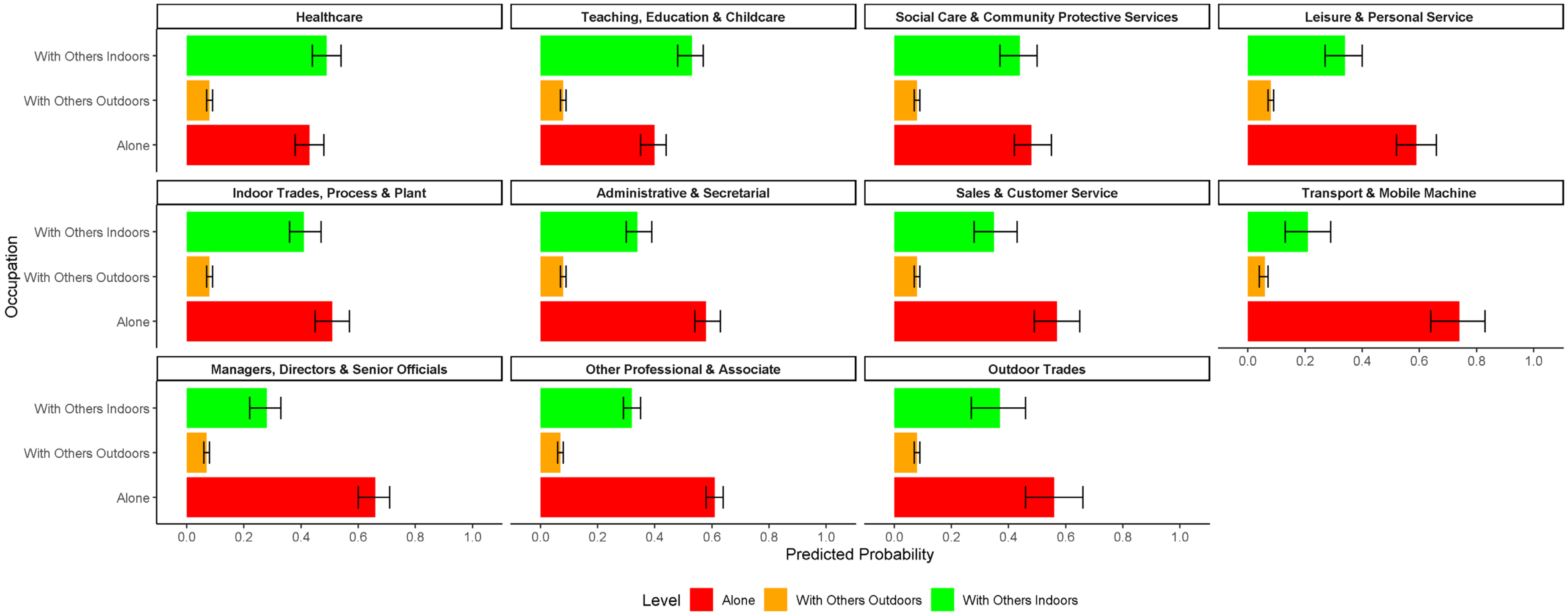

Note: Responses collected for whole survey period

Supplementary Figure S15. Precautions during Breaks Compared to Active Work: Predicted probabilities and 95% confidence intervals for reporting fewer precautions during breaks, by occupation (a) and time (b)

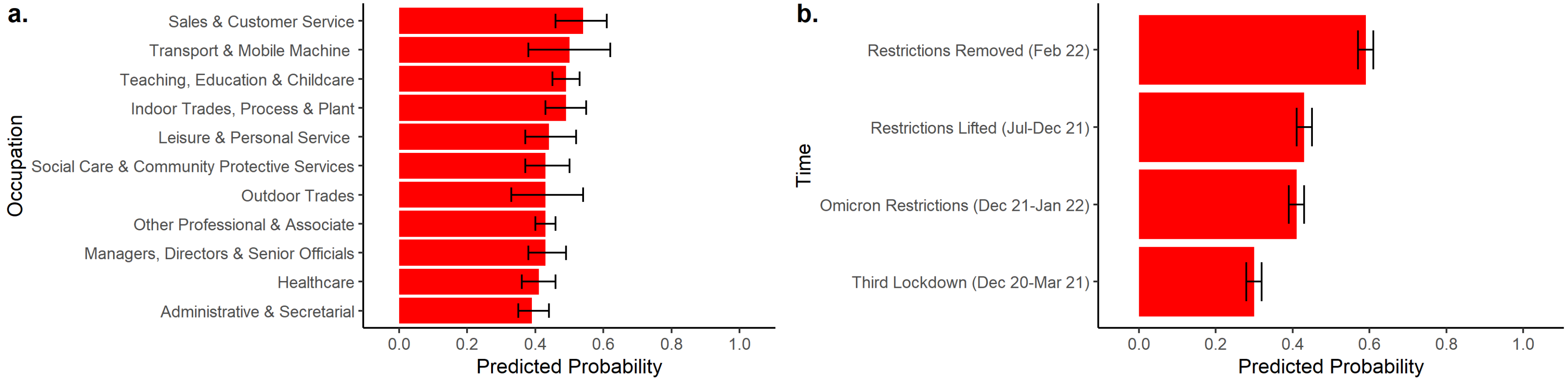

Note: Responses collected separately for major periods of restrictions; interaction term (occupation X period) not included based on Wald test

Supplementary Figure S16. Frequency of Work-Related Social Gatherings: Predicted probabilities and 95% confidence intervals for frequency levels, by occupation over time

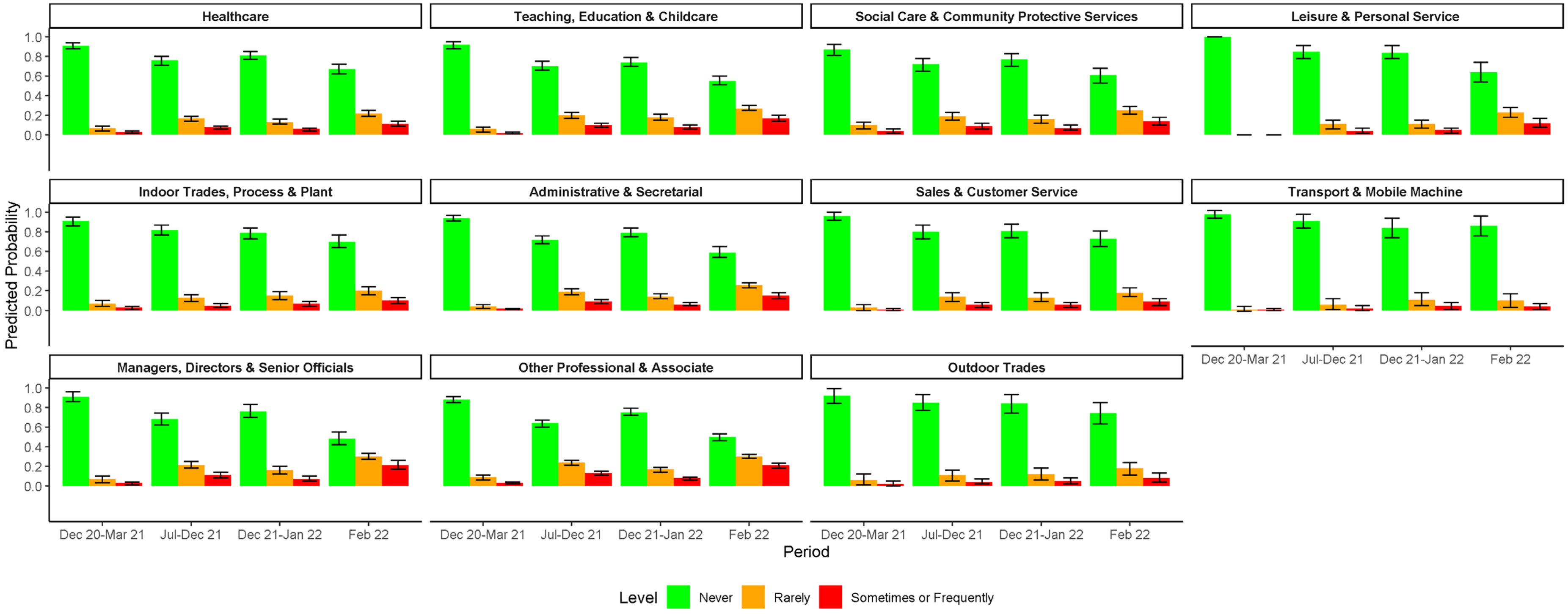

Note: Responses collected separately for major periods of restrictions; interaction term (occupation X period) included based on Wald test

Supplementary Figure S17. Workplace Provision of Lateral Flow Tests: Predicted probabilities and 95% confidence intervals for workplace ever providing testing, by occupation

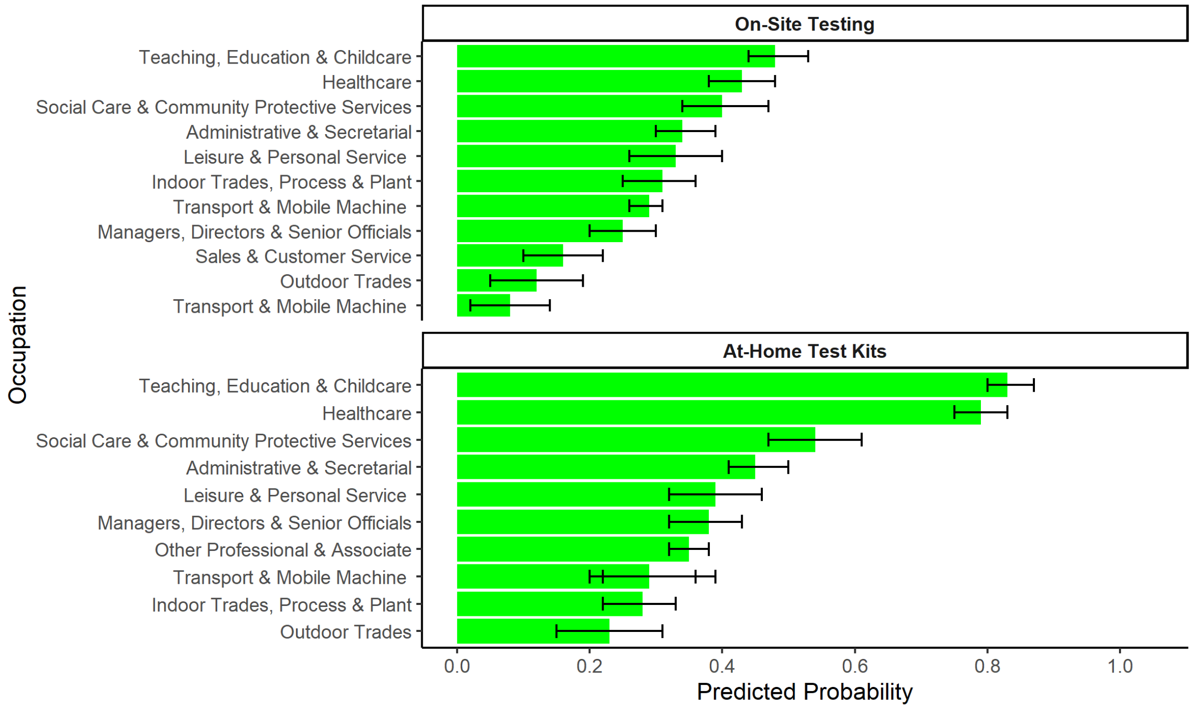

Note: Responses collected for whole survey period

Supplementary Figure S18. Perceptions of Work-Related Mitigation Methods during the Third National Lockdown: Proportions by occupation

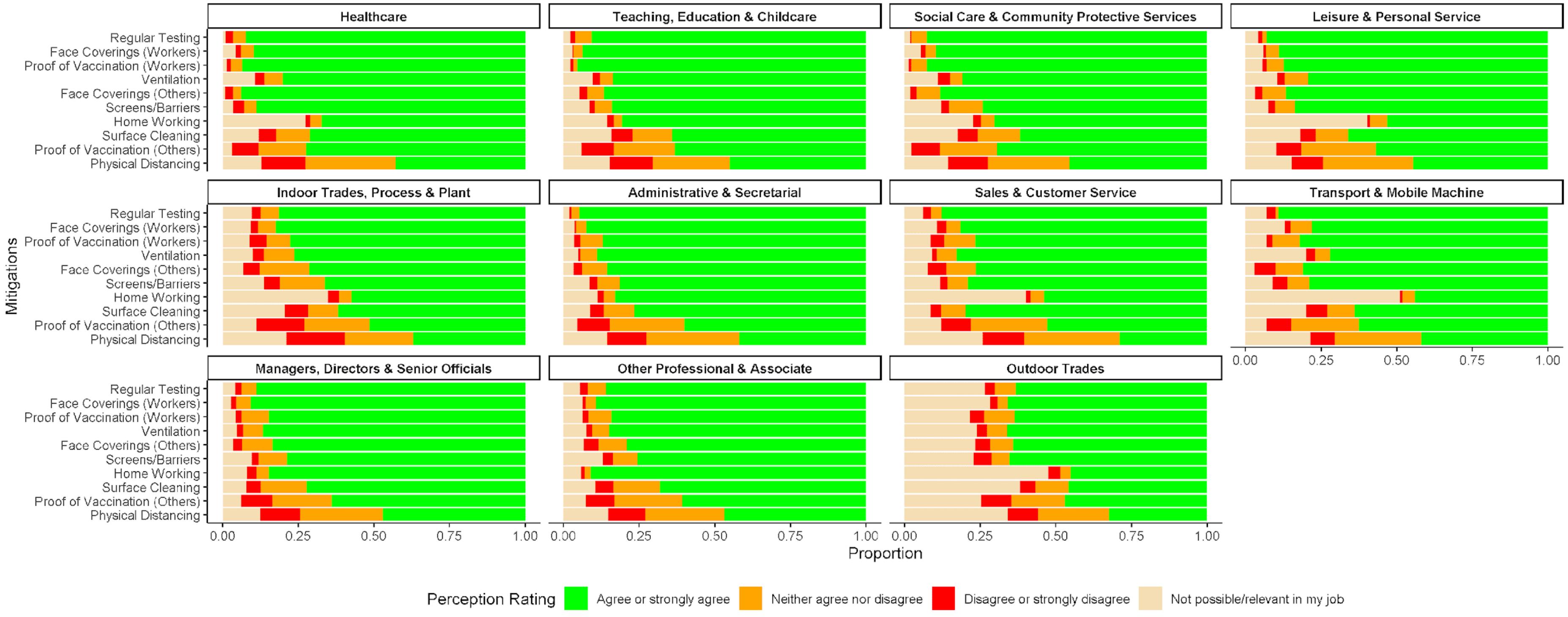

Note: Responses collected for single timepoint (third national lockdown)

Supplementary Figure S19. Perceptions of Work-Related Mitigation Methods in late February 2022: Proportions by occupation

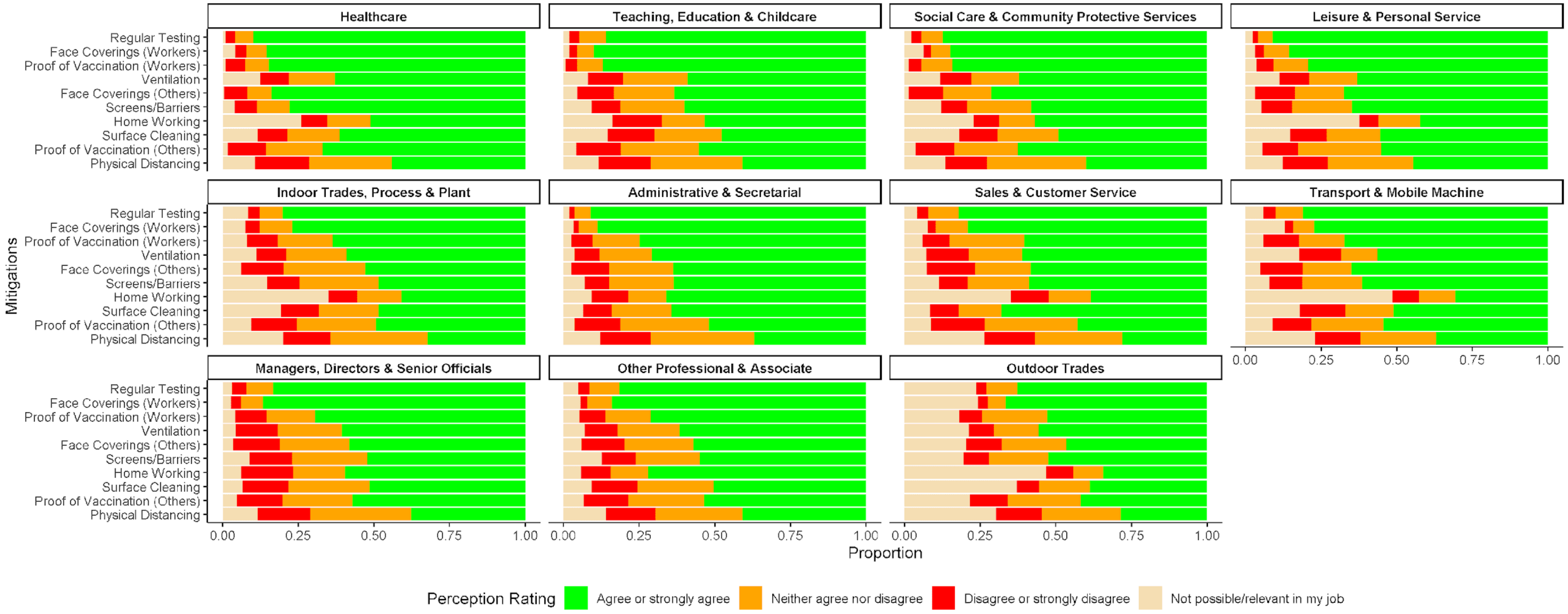

Note: Responses collected for single timepoint (late February 2022)
